# Bypassing Heteronuclear MRI: Metal‐Free Organosilicon Nanotracers for Background‐Free Hotspot ^1^H Imaging on Clinical Hardware

**DOI:** 10.1002/advs.77010

**Published:** 2026-08-06

**Authors:** Martin Orsagh, Dominik Havlicek, Andrea Galisova, Ondrej Sedlacek, Daniel Jirak

**Affiliations:** ^1^ Department of Physical and Macromolecular Chemistry Faculty of Science Charles University Prague Czech Republic; ^2^ Department of Diagnostic and Interventional Radiology Institute for Clinical and Experimental Medicine Prague Czech Republic; ^3^ Institute of Biophysics and Informatics First Faculty of Medicine Charles University Prague Czech Republic; ^4^ BIOCEV First Faculty of Medicine Charles University Vestec Czech Republic; ^5^ Faculty of Health Studies Technical University of Liberec Liberec Czech Republic

**Keywords:** magnetic resonance imaging, metal‐free nanotracers, nanoparticles, organosilicon polymers

## Abstract

Current magnetic resonance imaging (MRI) lacks contrast agents that provide background‐free hotspot visualization while remaining compatible with standard clinical hardware and avoiding metal‐associated toxicity. Herein, we introduce a new class of metal‐free, organosilicon‐based ^1^H MRI hotspot tracers that are directly compatible with conventional experimental and clinical MRI systems. These tracers exploit the distinct chemical shift of silicon‐shielded hydrogens (∼0 ppm) spectrally resolved from the biological ^1^H background. Through architectural screening, we identified an optimized block copolymer micelle, denoted as Silicone‐Generated Hotspot Tracer (SiGHT), comprising polydimethylsiloxane (PDMS) core and a hydrophilic shell. SiGHT exhibits excellent chemical and colloidal stability and enables sensitive, background‐free ^1^H chemical shift imaging at low concentrations (< 0.5 mg mL^−1^), while being non‐cytotoxic and well tolerated in vivo. The diagnostic potential of the developed probe has been demonstrated by successful ^1^H MR hotspot visualization of tracer accumulation in 4T1 mouse tumors following intravenous administration. Importantly, we demonstrate robust tracer detection on both preclinical 7T and clinical 3T MRI scanners using standard ^1^H MR sequences and commercial coils, underscoring its translational potential. Together, these results establish organosilicon micelles as clinically relevant, metal‐free ^1^H MR hotspot tracers, opening an unexplored pathway to accessible, high‐sensitivity molecular imaging on existing MRI infrastructure.

## Graphical abstract

Organosilicon micelles enable background‐free molecular magnetic resonance imaging (MRI) while remaining fully compatible with standard proton (^1^H) hardware. By exploiting a spectrally silent ^1^H window at ∼0 ppm, the silicone nanotracer allows hotspot detection by ^1^H chemical‐shift imaging (CSI) without metals or heteronuclei, enabling direct overlay with anatomical MRI on clinical scanners.

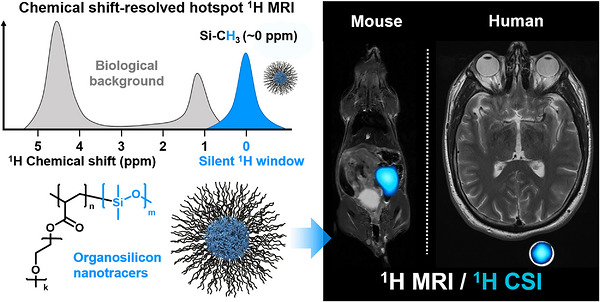

## 1 Introduction

Magnetic resonance imaging (MRI) is among the most widely used noninvasive non‐ionizing imaging techniques in both preclinical and clinical practice [1, 2]. Currently, hydrogen (^1^H) nuclei are most commonly used because of their high sensitivity and natural abundance in tissues, providing high soft tissue contrast. Generally, MRI contrast depends on tissue‐specific *T*_1_ and *T*_2_ relaxation times, which are affected by water/fat content and tissue composition. Differences in relaxation times enable the differentiation of various soft tissues, making MRI particularly valuable for anatomical imaging with exceptional spatial resolution [3].

However, in some cases, different tissues may exhibit similar relaxation times, making it difficult to differentiate them. To overcome this challenge, contrast agent tracers (CAs) are employed [4]. These compounds shorten the relaxation times of tissue hydrogens, thereby enhancing image contrast and facilitating the distinction of different anatomical structures. The most common CAs are paramagnetic gadolinium (Gd^3+^) complexes, which primarily shorten *T*_1_ relaxation times and produce hyperintense regions in *T*_1_‐weighted images [5, 6]. Alternatively, superparamagnetic iron oxide nanoparticles (SPIOs), which are often used for cell tracking, generate hypointense signals in regions surrounding SPIOs in *T*_2_‐weighted images [7, 8, 9]. Although clinically approved CAs are highly effective at improving sensitivity, their specificity remains limited, particularly when complex heterogeneous anatomical structures are being imaged. In such cases, determining whether the observed contrast originates from its intrinsic properties or from the administered CA itself may be challenging [10].

To overcome this specificity barrier, the concept of “hotspot” MRI has been introduced. Unlike conventional agents that merely alter surrounding tissue relaxation, hotspot imaging is based on detecting a direct, unambiguous signal originating exclusively from the tracer against a suppressed or zero biological background. Typically, non‐hydrogen MRI hotspot tracers have been developed in the preclinical setting, generating signals outside the biological background, providing such background‐free hotspot imaging. For instance, fluorine (^19^F) MRI is used because the human body contains negligible amounts of fluorine, thus avoiding interference from physiological signals [11, 12, 13]. Similarly, phosphorus (^31^P) MRI can be utilized, where the chemical shift of the phosphorus‐containing tracer is altered to avoid overlap with endogenous phosphorus signals [14, 15].

While non‐hydrogen MR methods offer highly specific tracer detection, their sensitivity is inherently lower than that of ^1^H MRI, and they often require specialized coils that are rarely available in the clinic [16, 17, 18]. As a result, no clinically used MRI contrast agents currently provide background‐free “hotspot” imaging while remaining compatible with standardized ^1^H hardware, representing a significant barrier to the widespread use of molecular MRI.

An emerging strategy is to design ^1^H‐based contrast agents whose resonances fall outside the physiological ^1^H spectrum, enabling selective detection with chemical‐shift‐resolved techniques such as ^1^H chemical shift imaging (CSI). In this “hotspot” approach, spectrally distinct tracer signals can be overlaid on anatomical ^1^H MR images, analogous to radiotracer imaging [19]. Recently, ^1^H CSI has been utilized to detect nanoparticles functionalized with polyethylene glycol (PEG), where the nanoparticles are used as hotspot contrast agents [20]. However, this method suffers from the spectral overlap of the PEG signal (∼3.7 ppm) with endogenous biological signals, which complicates in vivo imaging.

Several synthetic compounds with chemical shifts outside the physiological range, including organosilicon, imidazole, and thiazole derivatives, have already been reported [21]. However, these compounds have been rarely used for MR applications. Among these categories, organosilicon compounds have been the most widely explored [22, 23]. Nevertheless, owing to their lipophilicity and low water solubility, their application has been limited primarily to static oxygen concentration sensing at injection sites. The issue of low water solubility is commonly encountered in biomaterials design, and one main strategy to address this limitation is the attachment of solubilizing groups or the use of nanoparticles as drug carriers [24]. Nanoparticle systems offer numerous benefits, including the ability to avoid directly modifying the structure of the active compound while simultaneously enabling delivery to the site of need via passive or active targeting [25]. In this regard, polymer micelles stand out because of their simplicity and ease of modification, compared with clinically used small molecules such as Gd‐based contrast agents, where even small structural modifications can lead to prominent changes in properties.

**FIGURE 1:**
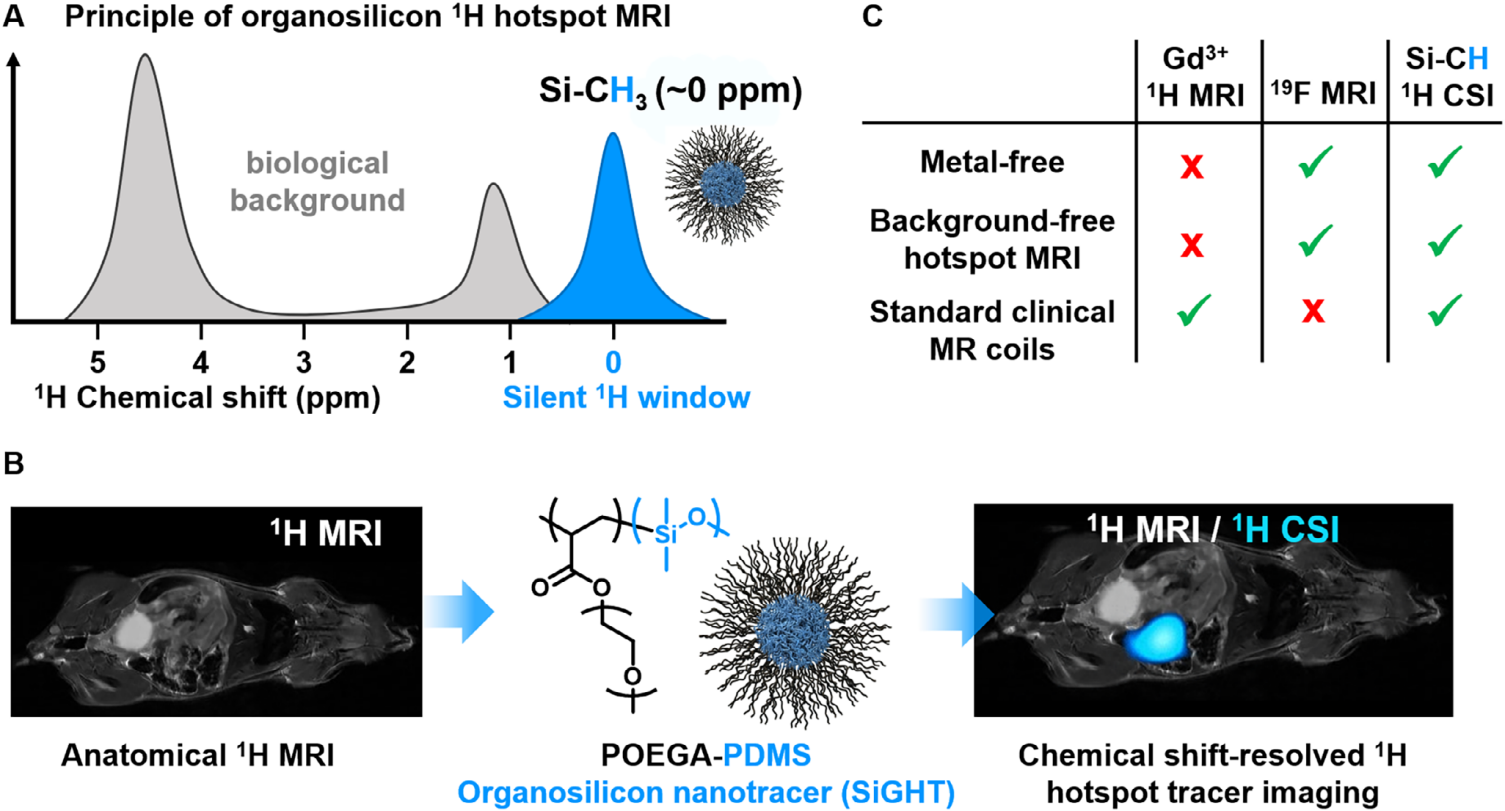
Schematic illustration of background‐free ^1^H MRI hotspot imaging using organosilicon micelles (SiGHT). Organosilicon micelles produce a spectrally isolated ^1^H signal at ∼0 ppm (A), enabling selective hotspot imaging by ^1^H chemical‐shift imaging (CSI) without endogenous background (B). Unlike gadolinium‐based contrast agents or ^19^F MRI, SiGHT provides background‐free detection while remaining fully compatible with standard ^1^H MRI hardware (C).

In this work, we designed and evaluated metal‐free, hydrophilized organosilicon tracers tailored for hotspot ^1^H CSI (Figure 1). From the library of different synthesized organosilicon architectures, we identified an optimized PDMS‐core, POEGA‐shell micelle (Silicone‐Generated Hotspot Tracer abbreviated as SiGHT) that combines (i) high organosilicon content and long *T*_2_ relaxation, (ii) excellent aqueous solubility and colloidal stability, and (iii) robust performance in both preclinical and clinical MRI settings. To our knowledge, SiGHT represents the first water‐soluble organosilicon‐based ^1^H MRI hotspot nanotracer demonstrated using a clinical MRI system, pointing to a realistic path toward metal‐free hotspot MRI with existing hardware.

## 2 Results and Discussion

In recent decades, organosilicon compounds (e.g., tetramethylsilane, TMS) have been used as reference standards for ^1^H NMR spectroscopy because of their very low characteristic MR chemical shifts (approximately 0 ppm) [26]. The low electronegativity of silicon causes a pronounced shielding effect in nearby MR‐active protons, making them spectrally resolvable from water and organic molecules present in the endogenous biological background. In this work, for the first time, we utilize the spectral isolation of organosilicon nanotracers to visualize their biodistribution by ^1^H MRI using ^1^H spectroscopic imaging. This enables frequency‐based selective detection and hotspot visualization, providing a more sensitive, metal‐free alternative to paramagnetic CAs (e.g., Gd^3+^). Furthermore, organosilicon compounds can be visualized with standard clinical ^1^H MR hardware, which provides an advantage over heteronuclear MR imaging (e.g., ^19^F MRI), which requires nonstandard MR coils. Unfortunately, the inherently nonpolar character of organosilicon compounds limits their potential for use in ^1^H MRI tracing applications.

**FIGURE 2:**
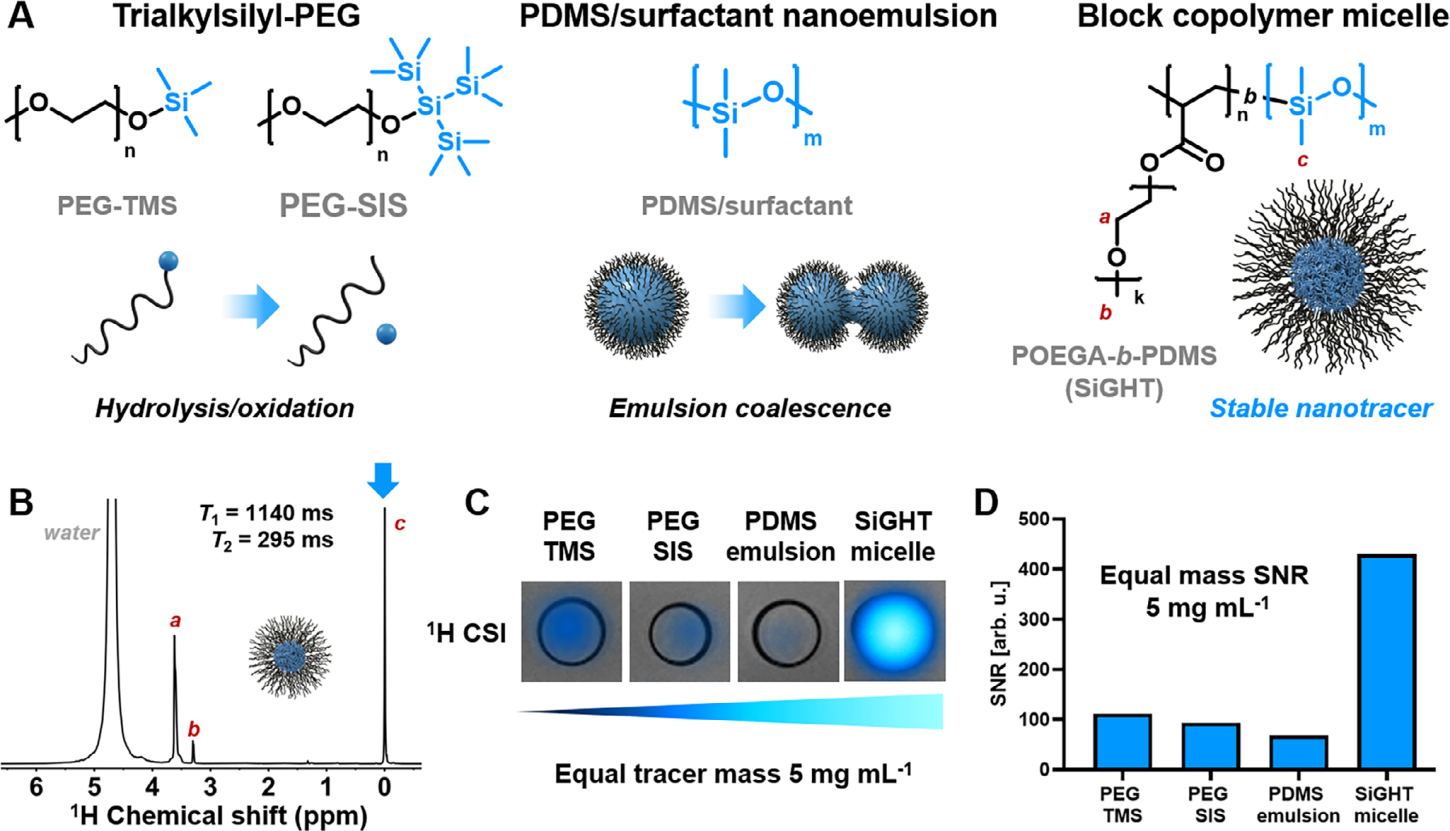
Architectural screening of organosilicon‐based ^1^H MRI tracers. (A) Comparison of representative organosilicon architectures evaluated in this work, highlighting their long‐term (in)stability. (B) Representative ^1^H NMR spectrum of POEGA‐*b*‐PDMS (SiGHT) block copolymer nanoparticles in water (D_2_O with 10% H_2_O), showing a resonance at ∼0 ppm that is spectrally separated from the common ^1^H background, relaxation times of hydrogens at 0 ppm. (C) In vitro phantom ^1^H CSI MRI at a constant tracer mass concentration (*c*_pol_ = 5 mg mL^−1^). (D) Quantitative signal‐to‐noise ratio (SNR) analysis confirming the enhanced ^1^H CSI performance of SiGHT relative to other organosilicon formulations.

We therefore pursued three complementary strategies to hydrophilize organosilicon tracers and systematically compared their performance in ^1^H CSI phantoms. First, we conjugated trialkylsilyl (i.e., trimethylsilyl and supersilyl) groups to water‐soluble poly(ethylene glycol) chains (Figure 2A). While the trimethylsilyl‐PEG ether exhibited good solubility, its hydrolytic instability led to significant degradation within several hours of incubation in water (Figure S13), as reported previously [27]. The supersilyl‐PEG derivative offers improved hydrolytic stability because of the steric bulkiness of the supersilyl group [28]; on the other hand, they exhibit lower solubility and significant aggregation, even at longer PEG chain length compared to PEG‐TMS, ultimately limiting signal intensity and practical usability. Furthermore, upon prolonged storage of the solid compound, a decrease in the signal at 0 ppm was observed (Figure S14), likely due to the hydrolytic, oxidative, or photolytic degradation of the supersilyl group [29, 30] followed by its aggregation in aqueous solutions.

We next turned our focus to polydimethylsiloxane (PDMS), a biocompatible silicone polymer with a low glass transition temperature (*T*_g_ ≈ ‐130°C) [31]. In its liquid‐like phase at room temperature, PDMS exhibits high chain mobility, resulting in sufficiently long *T*_2_ relaxation times of methyl hydrogens that can be detected by a conventional MRI scanner. Additionally, the high content of silicon‐bound protons in PDMS dramatically enhances the detection sensitivity. Similar emulsion systems have been reported as local oximetry sensors for Proton Imaging of Siloxanes to map Tissue Oxygenation Levels (PISTOL) [32]. Unfortunately, PDMS emulsions suffer from the leaching of organosilicones, limited colloidal stability, and limited payload loading per emulsion because of the high amount of surfactants required to stabilize the emulsions [32]. Therefore, we deemed this approach less suitable for in vivo use, particularly in comparison to the block copolymer approach mentioned below.

**FIGURE 3:**
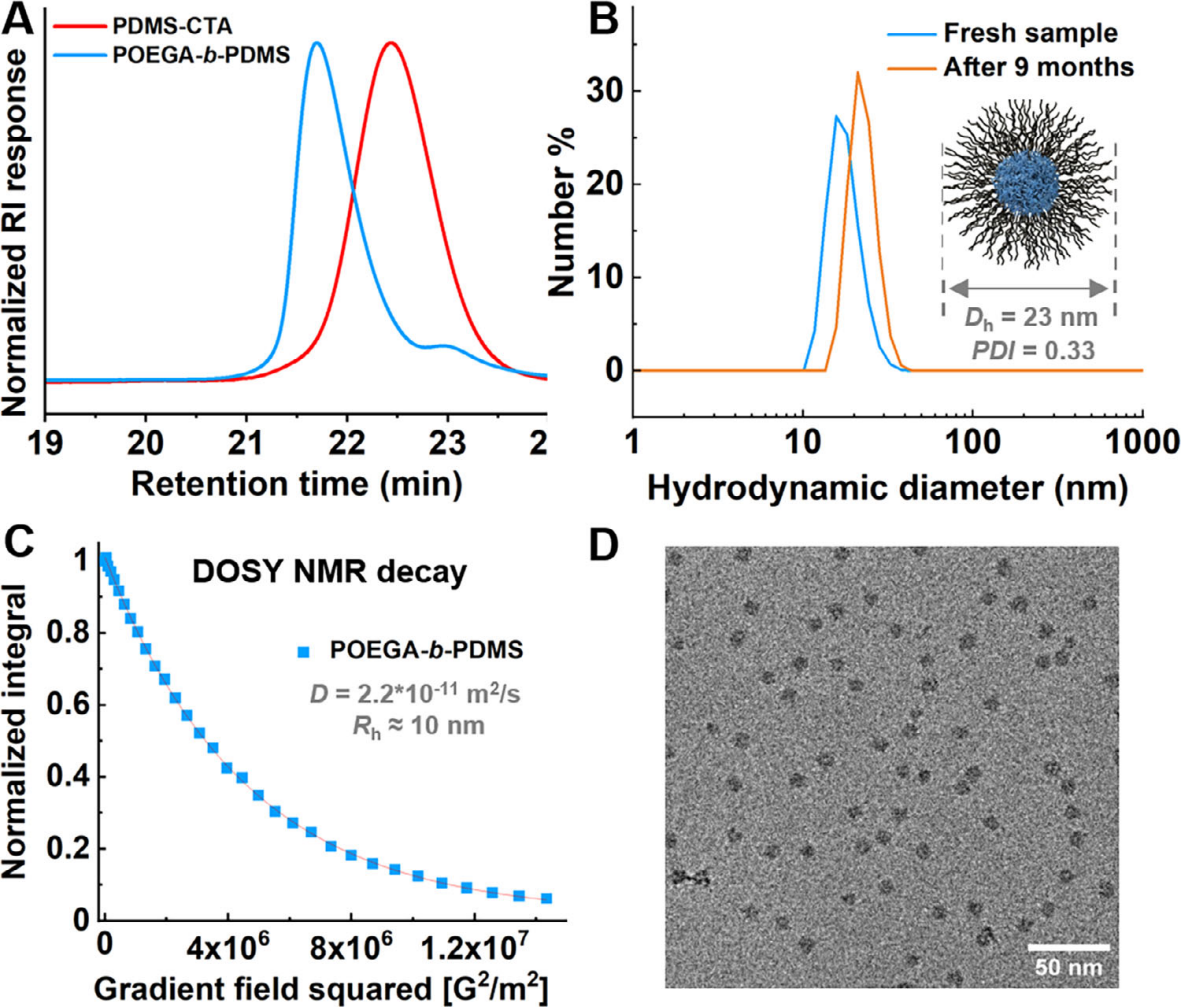
Physicochemical characterization of SiGHT. (A) SEC characterization of POEGA‐*b*‐PDMS (blue, SiGHT) prepared by chain extension of PDMS‐CTA (red). (B) Hydrodynamic diameter distribution of SiGHT, comparing freshly prepared sample (blue) and sample after 9 months storage at RT (unfiltered), measured by DLS (c_pol_ = 2 mg mL^−1^). (C) Dependence of the normalized integral of the PDMS signal in SiGHT micelle samples on gradient field strength in ^1^H DOSY NMR (measured in H_2_O with 10% D_2_O). (D) Cryo‐EM of SiGHT nanoparticles.

Finally, we designed amphiphilic diblock copolymers with a silicone‐rich PDMS hydrophobic block and a hydrophilic poly(PEG‐acrylate) block with a bottlebrush architecture (POEGA‐*b*‐PDMS; Figure 2A). This system combines the excellent MR properties of PDMS with outstanding stability. The copolymers were synthesized by blue‐light photoiniferter reversible addition‐fragmentation transfer (RAFT) polymerization [33], by chain‐extending the PDMS‐based chain transfer agent (PDMS‐CTA, Scheme S1), yielding well‐defined copolymers with low dispersity, high blocking efficiency, and a sharp ^1^H NMR signal at 0 ppm (Figures 2B and 3A). In water, these copolymers self‐assemble into stable micelles (termed SiGHT, for silicone‐generated hotspot tracer) that provide an organosilicon‐rich core for ^1^H CSI. Notably, both PDMS and PEG polymers are commonly used in bioengineering, and their derivatives are approved by the FDA for various clinical applications [34, 35, 36]. Furthermore, self‐assembled polymer nanoparticles can be used for encapsulation and delivery of hydrophobic drugs (i.e., theranostics) [37].

As a next step, we explored the MRI performance of the prepared organosilicon tracers in aqueous solutions to assess their suitability for ^1^H hotspot imaging. We conducted in vitro ^1^H chemical shift imaging (CSI) using Eppendorf tube phantoms containing four samples: PEG‐TMS, PEG‐SIS, PDMS emulsion, and POEGA‐*b*‐PDMS micelles in water. To more closely showcase practical usage scenarios, we prepared a series of samples with equal mass concentrations of each tracer (5 mg mL^−1^). Each sample (200 µL) was embedded in a 2% porcine skin gelatin matrix to mimic a biological background. Even though all compounds produced clear and distinguishable ^1^H CSI signals at 0 ppm, the POEGA‐*b*‐PDMS nanoparticles significantly outperformed the other investigated tracers in terms of signal intensity (Figure 2C,D). Considering their superior overall performance in ^1^H CSI and colloidal/chemical stability, the silicone‐based polymer micelles were then selected for further assessment.

Following the screening experiments, SiGHT micelles were subjected to detailed physicochemical characterization to assess their suitability as metal‐free ^1^H MRI hotspot tracers. In aqueous solution, the ^1^H organosilicon tracer formed narrowly dispersed nanoparticles with a hydrodynamic diameter of approximately 23 nm, as determined by dynamic light scattering (DLS, Figure 3B and Figure S15). A cryogenic transmission electron microscopy (cryo‐TEM) experiment confirmed a spherical morphology of the nanoparticles (Figure 3D), with sizes corroborating the DLS measurements. Furthermore, diffusion‐ordered spectroscopy (DOSY) NMR was used to estimate translational diffusion coefficients (Figure 3C), allowing hydrodynamic size estimation, which closely matched results from DLS/cryo‐TEM. The bottlebrush POEGA structure of the hydrophilic micellar shell enhanced both the water solubility of the polymer and the stability of the colloidal nanoparticles at high concentrations (up to 50 mg mL^−1^) by preventing aggregation. As a result, the micelles remained colloidally stable for at least several months at room temperature (Figure 3B), indicating a robust core‐shell structure suitable for biological environments.

**FIGURE 4:**
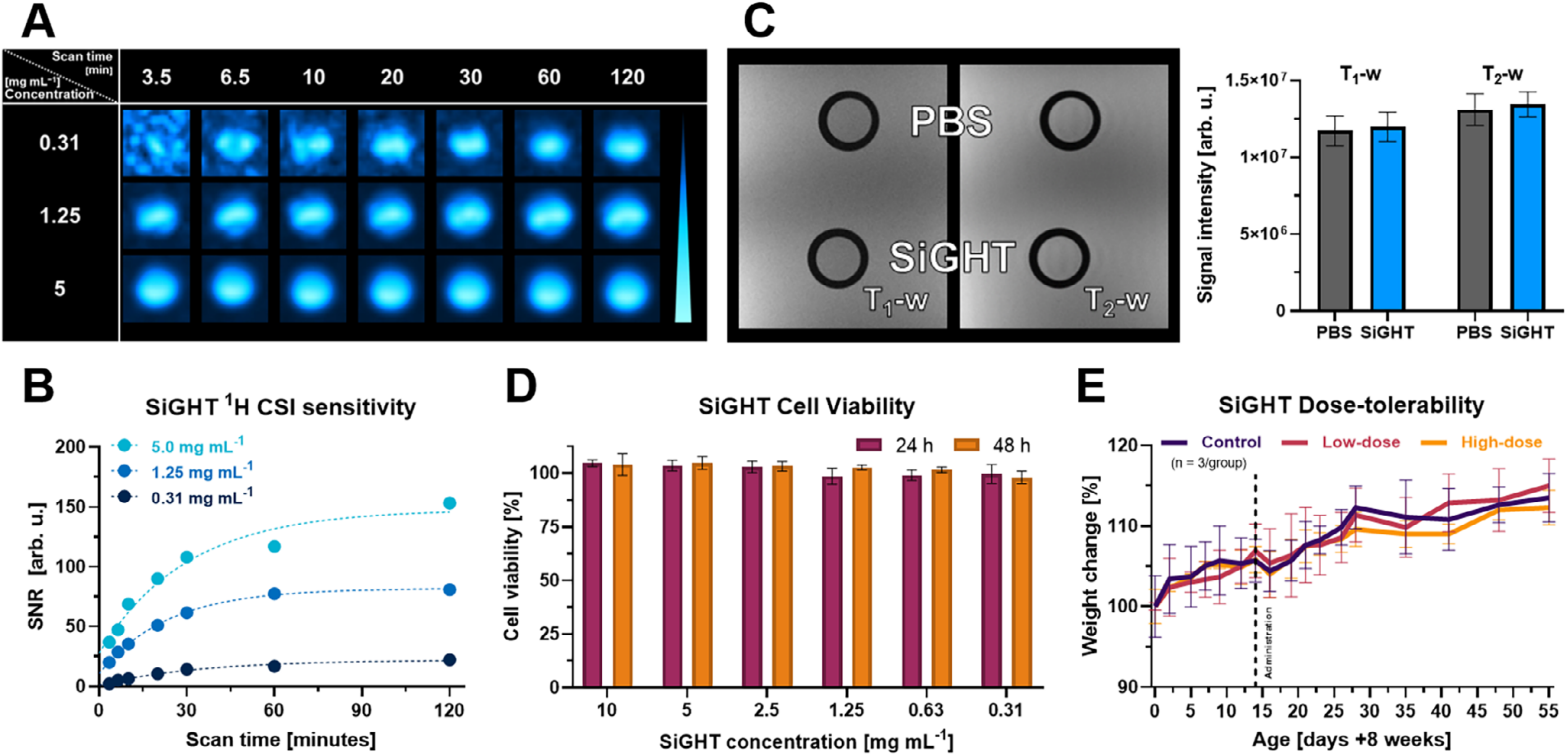
High ^1^H CSI sensitivity and biocompatibility of SiGHT. (A,B) Concentration sensitivity assessment of SiGHT using ^1^H CSI, demonstrating that SiGHT is detectable at sub‐mg mL^−1^ concentrations in a reasonable scan time. (C) Comparison of SiGHT and PBS in conventional ^1^H MRI in *T*_1_‐weighted (TR = 750 ms, TE = 11.5 ms) and *T*_2_‐weighted (TR = 3500 ms, TE = 60 ms) images shows no distinguishable contrast difference between SiGHT (5 mg mL^−1^) and PBS. (D) Absence of cytotoxicity of SiGHT in 4T1 cells. (E) Dose tolerability of SiGHT in healthy BALB/c mouse models (*n* = 3) after i.v. administration with SiGHT (*V* = 100 µL, c_pol_ = 10 or 50 mg mL^−1^), respectively PBS for the control group.

The high content of organosilicon groups within the liquid‐like PDMS core provided a sufficient amount of strongly shielded ^1^H atoms for hotspot generation. Notably, the tracer's *T*_2_ relaxation time (260 ms, as determined from 7T CSI images; 295 ms, as determined spectroscopically at 7T; Figures S19 and S20) was sufficiently long for effective signal acquisition under clinically relevant conditions. We then evaluated the visualization performance using ^1^H CSI. The micelles produced a strong, spectrally distinct hotspot signal at ∼0 ppm (Figure 2B), with no detectable overlap with the endogenous biological ^1^H background. The excellent ^1^H CSI tracer detection sensitivity was confirmed by measurements at varying polymer concentrations (Figure 4A,B). A distinct MR signal was detected at concentrations as low as 0.31 mg mL^−1^ within a reasonable scan time of 6.5 min at 7T, underscoring the high nuclei‐per‐mass capacity achievable because of the low atomic weight of hydrogen (Table S2). This high sensitivity highlights the tracer's suitability for low‐dose applications, particularly advantageous in biomedical contexts where tracer uptake is inherently limited, such as cellular labeling or systemic intravenous administration. Furthermore, to validate the tracer specificity and the reliability of the fat‐suppression strategy, we performed a targeted set of additional measurements. These included an in vitro gelatin phantom, a lipid‐rich ex vivo porcine model, and an in vivo subcutaneous injection experiment. Across these models, the organosilicon signal was selectively isolated from the endogenous background, with no detectable interference or substantial co‐suppression under the applied acquisition conditions (Figures S22–S24).

Notably, the tracer produces no detectable signal difference relative to a PBS phantom in gelatin in conventional *T*_1_‐ or *T*_2_‐weighted images, resulting in no interference with biological background MRI signals (Figure 4C and Figure S25).

To assess the tracer's suitability for biological applications, we first evaluated its cytocompatibility and interaction with mammalian cells. It exhibited minimal cytotoxicity toward 4T1 cells over a broad concentration range (0.16–10 mg mL^−1^), compared with the polymer‐free control cells, indicating excellent biocompatibility of the POEGA corona and the PDMS‐core micellar architecture (Figure 4D). Furthermore, the cellular uptake of SiGHT was studied by ^1^H CSI in 4T1 cells following 16 h of incubation. The MRI results confirmed successful internalization via endocytosis, with a clear, distinguishable signal observed in the ^1^H CSI images, whereas the nonlabeled control showed no detectable MR signal (Figure S26). These findings highlight the ability of the developed organosilicon nanoprobe to be effectively internalized by cells, opening up possibilities for cellular tracking in vivo.

In vivo biocompatibility was examined through intravenous administration in healthy BALB/c mice at two different doses (*c_pol_* = 10 or 50 mg mL^−1^, V = 100 µL, corresponding to a total dose of approximately 1 or 5 mg per mouse, respectively), which caused no observable adverse effects, behavioral changes, or abnormalities in body weight over the monitoring period (Figure 4E). These findings indicate that the POEGA‐*b*‐PDMS micelles are well tolerated in vivo and support the suitability of SiGHT as a metal‐free hotspot MRI tracer for systemic and longitudinal imaging studies.

**FIGURE 5:**
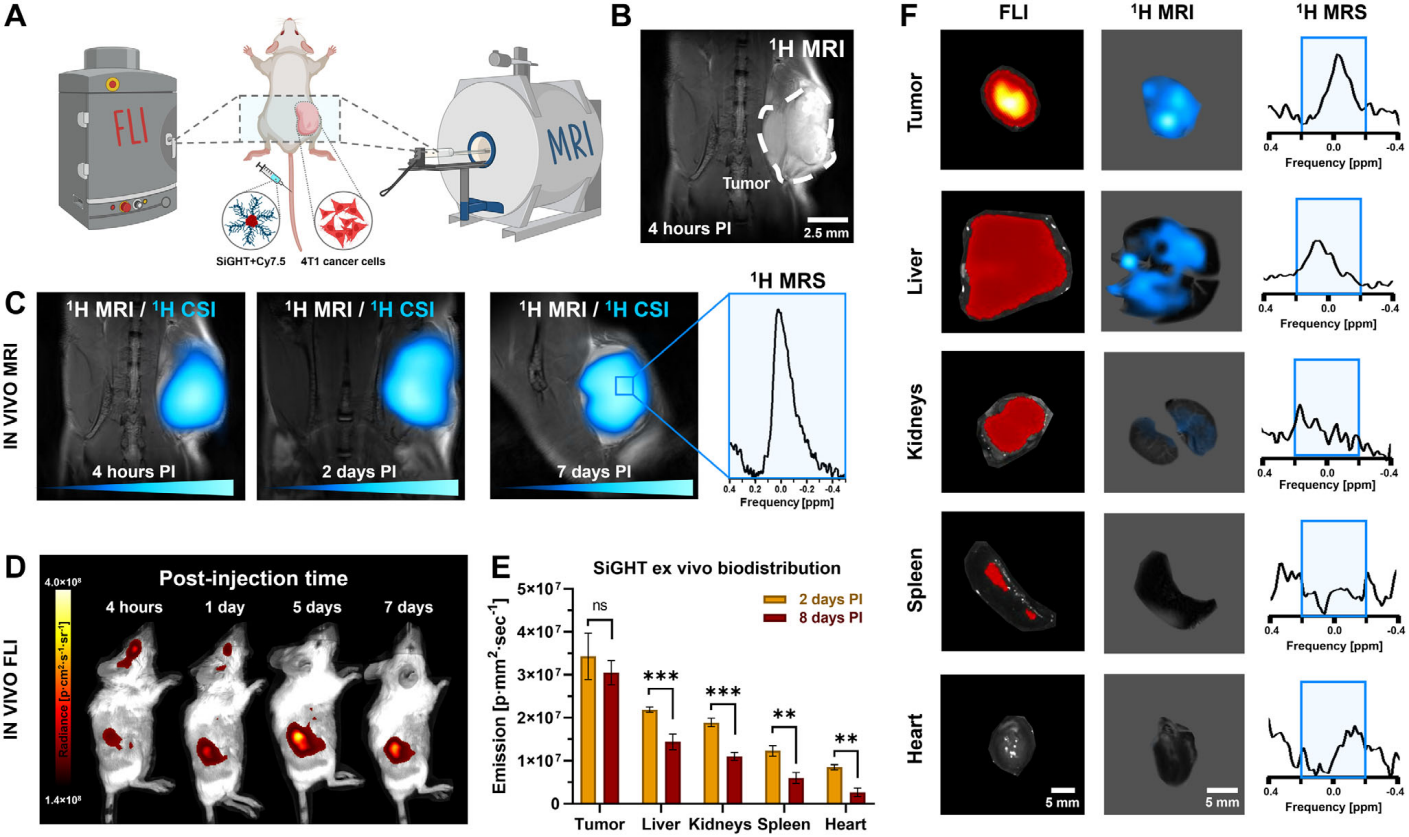
Silicone‐Generated Hotspot Tracer (SiGHT) visualization in biomedical settings. (A) To assess its in vivo/ex vivo distribution, SiGHT (*V* = 100 µL; *c*_pol_ = 50 mg mL^−1^) was injected intravenously into mice bearing subcutaneous 4T1 tumors and imaged using MRI/FLI. (B) *T*_2_‐weighted anatomical images of mice with tumor tissue highlighted. (C) Representative in vivo ^1^H CSI following the intravenous administration of SiGHT in tumor‐bearing mice, demonstrating tracer accumulation and retention in the tumor region over time. (D) Representative in vivo FLI images indicating accumulation and retention. (E) Semiquantitative FLI analysis revealing a statistically significant decrease in signal intensity at 8 days post‐injection (PI) compared with 2 days PI. (F) Representative ex vivo FLI images from vital organs show the highest tracer accumulation in the tumor, followed by the liver, and the same trend was observed using ex vivo ^1^H MRI and ^1^H MRS.

We next evaluated the potential for longitudinal hotspot imaging of 4T1 solid tumors in vivo (Figure 5A). Owing to its favorable hydrodynamic diameter (23 nm), SiGHT is well suited for passive accumulation in tumors, associated with the enhanced permeability and retention (EPR) effect [38], however, without absolute quantitative biodistribution data, we are unable to precisely determine the primary mechanism driving the tracer uptake in this model. For in vivo measurements, the voxel geometry for ^1^H CSI and the shimming volume were selected based on standard anatomical *T*_2_‐weighted MRI scans. Further adjustments were made by applying regular fat and broad water suppression pulses to reduce unnecessary signals in the spectral area of physiologically occurring signals while keeping the SiGHT signal at 0 ppm intact. Prior to tracer administration, no detectable signal at 0 ppm was observed in ^1^H CSI under the set acquisition parameters (Figure S27).

To demonstrate tumor imaging, the SiGHT nanotracer was intravenously administered to 4T1 tumor‐bearing mice (*V* = 100 µL, *c_po_*_l_ = 50 mg mL^−1^, *n* = 6). After 4 hours post‐injection (PI), a distinct signal at ∼0 ppm, corresponding to silicon‐proximal protons, became detectable within the tumor region, with no overlap with endogenous ^1^H signals (Figure 5C and Figure S28), confirming successful tracer accumulation. Furthermore, tracer accumulation in the tumor was confirmed by “background‐free” hotspot ^1^H CSI, which could be directly overlaid onto *T*_2_‐weighted anatomical images. A series of CSI imaging experiments was then performed at multiple time points up to 7 days PI, during which the tracer signal remained clearly detectable. The observed signal may reflect a combination of factors, including EPR‐related accumulation associated with tumor neovascularization, heterogeneous perfusion, vascular retention, and possible uptake by local phagocytic cells. The sustained signal over an extended time period highlights the stability and retention of the tracer within the tumor microenvironment, demonstrating the feasibility of the longitudinal monitoring of tracer dynamics.

While MRI has been employed as a primary imaging modality with clinical potential, the distribution of SiGHT tracers in tumor‐bearing mice has also been confirmed by fluorescence imaging (FLI). In contrast to MRI, the clinical relevance of fluorescence imaging is limited by light absorption and scattering in tissues [39]. Fluorescence imaging revealed progressive accumulation of the tracer with covalently attached sulfocyanine‐7.5 fluorophore within the tumor (Figure 5D), while the signal intensity in highly vascularized tissues and vital organs, such as the liver, steadily decreased over time. Semiquantitative analysis demonstrated an immediate dilution of the tracer within the body, peaking at 4 h PI before gradually decreasing, in line with the expected pharmacokinetics of nanoparticle‐based tracers [40]. The evaluation of the tumor‐to‐whole‐body fluorescence ratio further confirmed preferential retention within the tumor microenvironment over time (Figure S30).

The ex vivo distribution of fluorescently labeled SiGHT corroborates the ^1^H MRI experiments (Figure 5F), further validating the approach. Both MR and fluorescence imaging revealed the strongest signal from the tumor, confirming effective passive accumulation within the tumor microenvironment (Figure 5E,F and Figure S31). Notably, tumor‐associated fluorescence remained stable between day 2 and day 8 PI (p > 0.05), indicating prolonged retention. In contrast, fluorescence signals in clearance‐related organs, such as the liver and kidneys, decreased significantly over time (*p* < 0.001), reflecting the tracer's clearance from the systemic circulation. While circulating as stable micelles, the ultimate elimination pathway is expected to be renal filtration following the dynamic dissociation of the micelles into their block copolymer unimers, as these show molecular weights well below the renal clearance threshold (∼40 kDa). Short‐term biocompatibility was supported by histological examination upon hematoxylin and eosin staining of the spleen and kidney, which showed preserved tissue morphology and no apparent pathological alterations (Figure S32). Apart from the tumor uptake, the biodistribution was comparable between tumor‐bearing and control mice (p > 0.05), confirming that the pharmacokinetics were consistent regardless of tumor presence (Figures S30 and S31).

**FIGURE 6:**
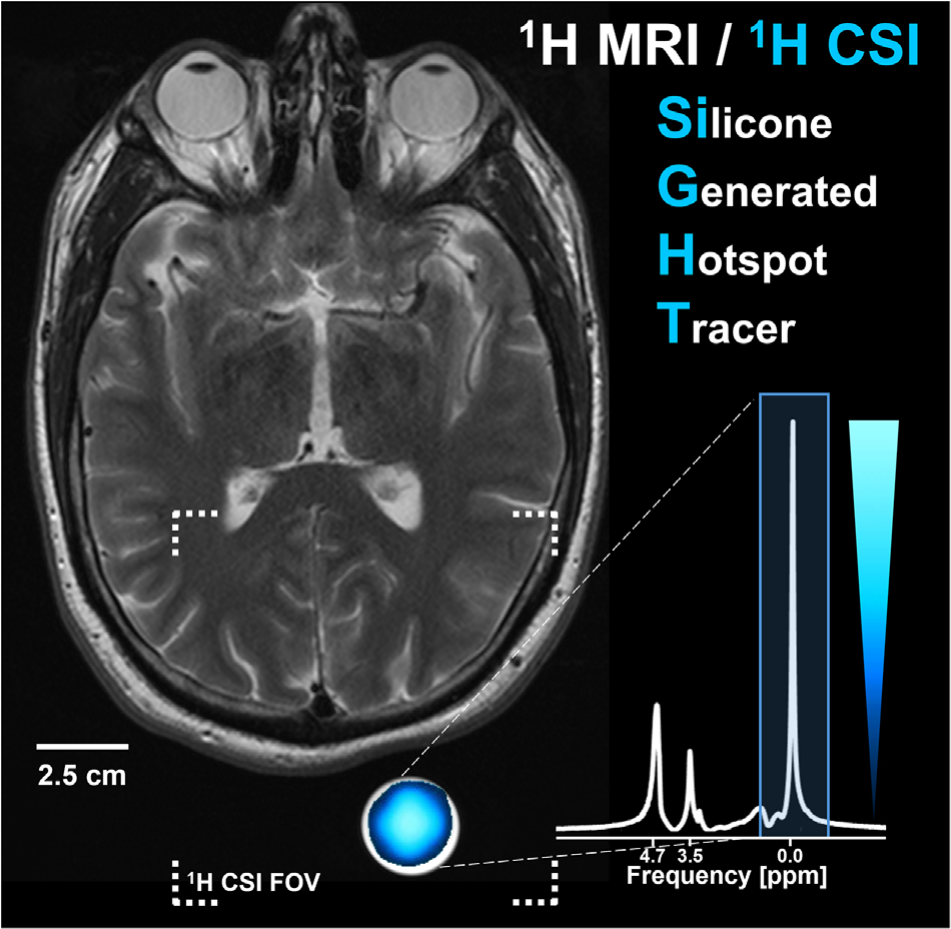
Axial cross‐section of the human brain with a SiGHT‐containing centrifuge tube phantom (*c*  = 20 mg mL^−1^) positioned behind the head. This proof‐of‐concept experiment demonstrates that SiGHT can be robustly detected in a clinical 3T setting using standard ^1^H CSI with a commercially available radio‐frequency coil.

Finally, to assess compatibility with clinically available instrumentation, we visualized the organosilicon tracer on a clinical 3T MRI scanner using a standard vendor‐supplied ^1^H CSI sequence and a commercially available head coil (Figure 6), following a typical ^1^H CSI protocol. As is typical for CSI acquisitions, the relatively long repetition times and low RF duty cycle resulted in low SAR values, remaining well below the limits defined by IEC 60601‐2‐33 Edition 4.0. A SiGHT‐containing tube phantom positioned adjacent to a human head produced a strong, spectrally distinct hotspot signal that could be cleanly overlaid on anatomical brain images, demonstrating that SiGHT can be detected with routine clinical hardware and sequences without any modification.

All animal protocols were approved by the Ethics Committee of the Institute for Clinical and Experimental Medicine and the Ministry of Health of the Czech Republic (No. 58/2014, Daniel Jirak) in accordance with the European Communities Council Directive (2010/ 63/EU).

## 3 Conclusion

In conclusion, this proof‐of‐concept study introduces a new class of metal‐free ^1^H MRI nanotracers based on organosilicon compounds that exploit the high colloidal stability and distinct chemical shift of silicon‐proximal hydrogens for hotspot imaging without interfering with conventional *T*_1_‐ or *T*_2_‐weighted images. The optimized micellar tracer, denoted as SiGHT, offers outstanding MRI sensitivity and spectral selectivity, providing a specific and more sensitive alternative to heteronuclear hotspot MR imaging and offering numerous opportunities for further development of this platform. Its potential for use in both diagnostic and therapeutic monitoring applications, ranging from short‐term assessments to long‐term tracking, makes SiGHT an intriguing option for possible future biomedical applications. Incorporation of therapeutic payloads or functionalization with targeting ligands could enable tissue‐specific delivery, allowing these ^1^H‐traceable contrast agents to serve as both drug delivery vehicles and imaging probes for longitudinal treatment monitoring.

## Author Contributions

**Dominik Havlicek**: conceptualization, investigation, data curation, validation, formal analysis, writing – original draft. **Andrea Galisova**: methodology, formal analysis, investigation. **Martin Orsagh**: conceptualization, methodology, investigation, validation, formal analysis, writing – original draft. **Daniel Jirak**: methodology, data curation, validation, formal analysis, supervision, funding acquisition, visualization, project administration, resources, writing – review and editing. **Ondrej Sedlacek**: methodology, investigation, validation, supervision, funding acquisition, visualization, project administration, resources, writing – review and editing.

## Conflicts of Interest

M.O., D.H., O.S., and D.J. disclose the filing of a Czech patent N° D25099273 (PV 2025–432) concerning the topic of this manuscript (application date: 09/25/2025). The other author has no conflicts to declare.

## Supporting information




**Supporting File**: advs77010‐sup‐0001‐SuppMat.pdf.

## Data Availability

The data that support the findings of this study are available from the corresponding author upon reasonable request.
