# Supplementary material for: Bypassing Heteronuclear MRI: Metal‐Free Organosilicon Nanotracers for Background‐Free Hotspot 1H Imaging on Clinical Hardware
Source: Adv Sci (Weinh). 2026 Aug 6;13(60):e77010. doi: 10.1002/advs.77010 (PMC13445333; doi:10.1002/advs.77010)
Supplement: Supplementary file 1 [file ADVS-13-e77010-s001.pdf]

Supporting Information

Bypassing Heteronuclear MRI: Metal-Free Organosilicon Nanotracers for Background-Free Hotspot ^{1}H Imaging on Clinical Hardware

Martin Orságh,[a,†] Dominik Havlicek,[b,c,†] Andrea Galisova,[b,d] Ondrej Sedlacek,[a,*] Daniel Jirák[b,c,e,*]

[a]Department of Physical and Macromolecular Chemistry, Faculty of Science, Charles University, Hlavova 8, 128 00 Prague 2, Czech Republic

[b]Department of Diagnostic and Interventional Radiology, Institute for Clinical and Experimental Medicine, Prague 140 21, Czech Republic

[c]Institute of Biophysics and Informatics, First Faculty of Medicine, Charles University, Prague 2, Prague, 128 00, Czech Republic

[d]BIOCEV, First Faculty of Medicine, Charles University, Prumyslova 595, 252 50 Vestec, Prague region, Czech Republic

[e]Faculty of Health Studies, Technical University of Liberec, Studentská 1402/2, 46117 Liberec, Czech Republic

[†]Equally contributing authors

*email: daniel.jirak@ikem.cz; sedlacek@natur.cuni.cz

Experimental Section

Materials and Methods

All chemicals were purchased from Sigma-Aldrich (Prague, Czech Republic) unless stated otherwise (including monohydroxy-terminated polydimethylsiloxane (PDMS, M_n = 4670 g mol^{-1})). Polyethylene glycol monomethyl ether (M_n = 440 g mol^{-1} and M_n = 4,000 g mol^{-1}) and tris(trimethylsilyl)silyl chloride were purchased from TCI. Dried dichloromethane (DCM) over molecular sieves (AcroSeal) used for the synthesis of PEG-TMS and PEG-SIS was purchased from Thermo Fisher Scientific. Common laboratory solvents were purchased from Lachner and used without further purification unless otherwise stated. Ethyl acetate used for the synthesis of fluorescently labeled SiGHT (POEGA-co-SCy7.5Am-b-PDMS) has been dried over molecular sieves 3Å. Sulfo-Cyanine7.5 amine has been purchased from Lumiprobe (Germany). 2-(Butylthiocarbonothioylthio)propanoic acid (BTPA) chain transfer agent was synthesized according to the literature procedure.[1] Dialysis membranes (Spectra/Por MWCO 3.5 kDa and 7 kDa) were purchased from Roth (Germany). All other chemicals were purchased from Sigma-Aldrich unless stated otherwise.

Synthesis of PEG-TMS:

MeO-PEG-OH (500 mg, 1.14 mmol, M_n = 440 g mol^{-1}) and triethylamine (180 µL, 1.29 mmol) were dissolved in dry tetrahydrofuran (THF, 2 mL) in an 8 mL glass vial. Trimethylsilyl chloride (160 µL, 1.26 mmol) was added dropwise under vigorous stirring. The reaction mixture was stirred at room temperature for 6 hours. Afterwards, the reaction mixture containing the white precipitate was diluted with 5 mL of THF and filtered to remove the triethylamine hydrochloride precipitate. The filtrate was concentrated under reduced pressure and further dried under vacuum, yielding pure PEG-TMS (520 mg, 89% yield, 90% chain-end functionalized) as a transparent, colorless, viscous liquid. The structure of PEG-TMS was confirmed by ^{1}H and ^{13}C NMR (**Figure S1, S2**)

Synthesis of PEG-SIS:

In a 4 mL glass vial, MeO-PEG-OH (M_n = 4,000 g mol^{-1}, 600 mg, 0.15 mmol) and tris(trimethylsilyl)silyl chloride (100 mg, 0.35 mmol)were dissolved in 2 mL of dry DCM. With constant stirring, a solution of dimethylaminopyridine (DMAP, 80 mg, 0.66 mmol) in DCM (0.5 mL) was added dropwise to the reaction mixture, which was stirred for 48 hours. The crude product contaminated with DMAP residue was obtained by precipitation with hexane, isolated by centrifugation, and further washed with hexane. DMAP residue has been removed by redissolving the crude product in 4 mL of THF and centrifuging off undissolved solids. After evaporating the THF supernatant, pure PEG-SIS as a solid white powder has been obtained (440 mg,

69 % yield, 70% functionalized). The structure of PEG-TMS was confirmed by ^{1}H and ^{13}C NMR (**Figure S3, S4**)

Preparation of PDMS emulsion:

Monohydroxy-terminated PDMS (M_n = 4670 g/mol, 300 mg, 0.064 mmol) and Tween 80 surfactant (1.2 g, 0.91 mmol) were thoroughly mixed in a 15 mL centrifuge Falcon tube, followed by the addition of water to a total volume of 10 mL. The mixture was homogenized for 5 minutes at room temperature using an IKA Ultra-Turrax T-18 homogenizer with a 10 mm S5 dispersing tool, operating at 10,000 rpm, no more than 15 minutes before each experiment (see **Figure S5** for the ^{1}H NMR spectrum).

Synthesis of PDMS-CTA (PDMS-BTPA):

In a 100 mL round-bottom flask, PDMS-OH (10.0 g, M_n = 4670 g/mol, 2.1 mmol) and BTPA (1.05 g, 4.4 mmol) were dissolved in dry DCM (20 mL). The reaction mixture was cooled in an ice bath, and a solution of *N*, *N'*-dicyclohexylcarbodiimide (DCC, 1.10 g, 5.3 mmol) and DMAP (15 mg, 0.12 mmol) dissolved in dry DCM (15 mL) was added slowly by syringe pump over 30 minutes with constant stirring. The reaction mixture was then allowed to warm to room temperature and left stirring for 3 days. Afterwards, 100 µL of water was added to the reaction mixture, which was stirred for an additional 2 hours. The DCM was evaporated under reduced pressure, and the resulting oil was redissolved in 150 mL of hexane. The reaction mixture was washed with methanol (4 x 200 mL), distilled water (2 x 150 mL), and dried over anhydrous $MgSO_4$. Hexane was removed under reduced pressure, yielding 9.2 g of yellow oily product (87% yield). The polymer was characterized by ^{1}H NMR (**Figure S7**) and SEC (**Figure S8** and **Table S1**).

Synthesis of POEGA-b-PDMS (SiGHT):

In a 4 mL glass vial, polydimethylsiloxane (PDMS) chain-end modified with 2-(butylthiocarbonothioylthio)propanoic acid chain transfer agent (PDMS-CTA, **Scheme S1**, 155 mg, 31.7 µmol) and 270 mg of oligoethylene glycol acrylate (OEGA, M_n = 480 g mol^{-1}, 0.56 mmol) were dissolved in 2.1 mL of dried ethyl acetate. The vial was sealed and purged with nitrogen for 20 minutes to remove any dissolved oxygen. The vial was then exposed to LED blue light irradiation (450 nm, 25 mW cm^{-2}) for 18 hours. Ethyl acetate was removed on rotavapor, and the polymer was redissolved in 2 mL of tetrahydrofuran (THF). The polymer solution was diluted with 2 mL of water and dialyzed against water for 3 days (molecular weight cut-off, MWCO = 3.5 kDa), yielding the micellar solution. The micellar solutions were subsequently concentrated by ultracentrifugation using Amicon tubes (MWCO 3 kDa). Anhydrous polymers were obtained by lyophilization from water. The copolymer structure has been confirmed by size exclusion chromatography (SEC, **Figure 3A**, **Table S1**) and NMR spectroscopy (**Figure S8-S10**).

Synthesis of POEGA-*co*-SCy7.5Am-*b*-PDMS (fluorescently labeled SiGHT):

In a 4 mL glass vial, PDMS-CTA (155 mg, 31.7 µmol), OEGA (M_n = 480 g mol^{-1}, 270 mg, 0.56 mmol), and pentafluorophenyl acrylate (1.0 mg, 4.2 µmol) were dissolved in dry ethyl acetate (2.1 mL). The vial was sealed and purged with nitrogen for 20 minutes to remove any dissolved oxygen. The vial was then irradiated with LED blue light (450 nm, 25 mW) for 18 hours. Aliquots for NMR and SEC were taken. The reaction mixture was then concentrated under an inert atmosphere to a total volume of 1 mL, and a solution of SulfoCyanine-7.5-amine (5.5 mg, 4.8 µmol), triethylamine (5 µL, 40 µmol), and 1-Ethyl-3-(3-dimethylaminopropyl)carbodiimide hydrochloride (EDC, 2 mg, 10 µmol) in dry degassed dimethyl formamide (DMF, 3 mL) was added. Ethyl acetate was removed from the reaction mixture by rotary evaporation, and the solution was stirred for 3 days at room temperature (reaction progress monitored by SEC-UV at 770 nm). After the reaction was completed, 3 mL of water was added to the reaction mixture, and the mixture was dialyzed into water for 3 days (MWCO 3.5 kDa), followed by an additional 2 days (MWCO 7 kDa). This approach yields micelles of pure POEGA-*co*-SCy7.5Am-*b*-PDMS; the micellar solutions were subsequently concentrated by centrifugation using Amicon tubes (MWCO 3 kDa). Purified POEGA-co-SCy7.5Am-b-PDMS was isolated by lyophilization of the micellar solution. The content of Cy7.5 in the copolymer has been determined by UV/vis spectrometry (SHIMADZU UV-2401PC) at 778 nm (ε = 222,000 L mol^{-1} cm^{-1}).

Characterization of synthesized tracers

Size exclusion chromatography (SEC) was performed using an Agilent 1260 Infinity system with an RI detector (G1362A, thermostated at 35 °C). The separation was performed on two PLgel (Agilent) columns: Mixed C 300 x 7.5 mm, 5 µm, and Mixed D 300 x 7.5 mm, 5 µm, in series, thermostatted at 30°C in tetrahydrofuran at an elution rate of 0.7 mL/min. Molar masses and dispersities were calculated against narrow-dispersity poly(styrene) standards (Polymer Laboratories), M_p = 1950 – 89400 g mol^{-1}. For monitoring fluorescent probe attachment during the synthesis of fluorescently labeled SiGHT via SEC-UV/Vis, the Watrex Streamline system, equipped with a Streamline P1 pump, a Streamline AS2 autosampler, a Streamline CT Column Thermostat, a Streamline UV detector, and a Streamline RI detector, was used. The separation was performed on two PLgel 5 µm mixed-D columns in series, thermostatted at 55 °C in *N,N*-dimethylacetamide (DMAc) containing 50 mM LiCl, at an elution rate of 0.5 mL min^{-1}.

^{1}H and ^{13}C NMR spectra were recorded on a Bruker Avance MSL 400 MHz and Bruker Avance Neo 400 MHz spectrometer at room temperature in $CDCl_3$ or H_2O/D_2O mixtures (unless stated otherwise). The T_1 and T_2 relaxation times were measured using Varian VNMRS 300 MHz (7 T) via Inversion Recovery and CPMG sequences, respectively. For both relaxation time measurements, a relaxation delay of 10 s and an

acquisition time of 1.7 s were used; number of scans = 4. For the CPMG measurement, the value of $2\tau = 2$ ms was used.

T1: the inversion recovery delays listed in seconds:
0.0625, 0.1017, 0.1655, 0.2693, 0.43823, 0.7131, 1.1604, 1.88826, 3.07267, 5.

T2 measurement CPMG evolution times (relaxation delays) listed in seconds:
0.025, 0.03966, 0.06291, 0.09978, 0.15828, 0.25108, 0.39828, 0.63177, 1.00216, 1.58968, 2.52165, 4

^{1}H DOSY NMR spectra for the POEGA-*b*-PDMS (SiGHT) and POEGA-co-SCy7.5Am-b-PDMS (fluorescently labeled SiGHT) were recorded using a Bruker Avance III 600 MHz spectrometer equipped with a cryogenically cooled probe. ^{1}H DOSY measurements were performed in H_2O with 10% of D_2O (v/v) with a double-stimulated echo experiment with bipolar pulse field gradients using 32 gradient values linearly spaced between 2% and 98% of the maximal value (50 G cm^{-1}).[2] The hydrodynamic radius was estimated assuming the viscosity of pure H_2O (1.0 mPa s) at a temperature of 25 °C.

Critical micelle concentration (CMC) was determined by fluorescence spectrometry (Fluorolog FL 3–22 fluorimeter, Horiba Jobin Yvon, France). A stock solution of 0.5 mg mL^{-1} Nile red in 50% methanol (3 μL) was added to each sample of a series of micellar solutions (2.5 mL each) of different polymer concentrations. After incubation for 24 h at room temperature, fluorescence was measured using an excitation wavelength λ_{ex} = 550 nm. The CMC value was determined as the range at which the emission maximum of NR shifts from ~630 nm (encapsulated NR) to ~660 nm (free NR), as well as from a sudden increase in fluorescence intensity at 630 nm.

Dynamic light scattering (DLS) was used to determine the hydrodynamic diameter of the SiGHT copolymer nanoparticles using a ZEN3600 Zetasizer Nano-ZS analyzer (Malvern Instruments, UK). The samples were filtered through a 0.22 μm PTFE syringe filter unless otherwise stated. The apparent hydrodynamic diameter of the nanoparticles (D_h) and their polydispersity index (PDI) were determined at a scattering angle of θ = 173°. The data were analyzed using the DTS (Nano) program. For the stability experiment, aqueous stock solution of SiGHT (20 mg mL^{-1}) was stored for 9 months at room temperature, followed by dilution to 2 mg mL^{-1} for DLS measurement.

For *cryo-transmission electron microscopy* (cryo-TEM), 4 μL of sample (10 mg mL^{-1}) was applied to a glow-discharged copper cryo-EM grid (Quantifoil R 1.2/1.3, 300 mesh) and incubated for 10 seconds at room temperature and 80% relative humidity in a Leica GP2 Plunge Freezer. The grid was then automatically blotted using filter paper for 4 s and immediately vitrified by plunging into liquid ethane cooled to −180 °C. After vitrification, the sample was transferred to a dual-grid cryo-holder from Simple Origin and inserted into the transmission electron microscope (TEM).

Cryo-TEM data were acquired in a semi-automated mode using SerialEM on a JEOL JEM-2100Plus TEM equipped with a LaB_6 electron gun and a TVIPS XF416 CMOS camera, operated at 200 kV.[3] Images were acquired at 80,000× magnification (pixel size of 0.143 nm) with a 3 s exposure time, defocus values ranging from −3.0 to −2.0 μm, and a total electron dose of 50 $e^-/Å^2$ per image.

Cytotoxicity assay

The mouse breast cancer cell line 4T1 was used for the experiments. The cells were cultured in RPMI medium supplemented with 10% fetal bovine serum and 1% penicillin/streptomycin (Thermo Fisher Scientific, USA) and regularly checked for mycoplasma contamination. For the cytotoxicity examination, the cells were seeded in 96-well plates (7,000 cells/well) in complete RPMI medium and incubated overnight at 37 °C with 5% CO_2. The following day, cells were supplemented with the POEGA-b-PDMS tracer at serially diluted concentrations: 10, 5, 2.5, 1.25, 0.62, 0.31, and 0.15 mg mL^{-1}. Untreated cells served as negative controls. After 24 h and 48 h of incubation, the medium was carefully removed, and the cells were washed four times with phosphate-buffered saline (PBS). Alamar Blue reagent (Thermo Fisher Scientific, USA) (10-times diluted in complete RPMI medium) was then added to each well (100 μL/well), followed by a 4-hour incubation at 37 °C. Fluorescence (excitation at 540 nm, emission at 590 nm) was then measured using an ELISA Microplate Reader RT-6900 (Gen5 Software, BioTek, Germany), and cell viability was calculated.

Cellular uptake

4T1 cells (passage 19) were seeded in 6-well plates and incubated overnight at 37 °C with 5% CO_2 in the full RPMI culture medium (10% FBS, 1% penicilin/streptomycin). The following day, cells were treated with the POEGA-*b*-PDMS tracer at a concentration of 10 mg mL^{-1}. Control (non-labeled) cells received no compound. After 24 hours of incubation, the medium was removed, the cells were washed three times with PBS, detached using trypsin (incubation for 7 minutes at 37 °C), and collected by centrifugation at 200 × g for 5 minutes. The cell pellets were resuspended in 4% paraformaldehyde (PFA) and incubated for 20 minutes at room temperature. Following fixation, the cells were washed twice with PBS, and the final pellets were transferred to tubes for MRI analysis.

To confirm cellular uptake further, 100 μL of labeled and unlabeled cellular suspension was diluted with 500 μL of 0.9% NaCl in D_2O. The samples were transferred to cuvettes, left to freely sediment for 15 minutes, and the ^{1}H NMR with presaturation at the water signal was acquired. Upon cell lysis (after adding 20 μL of 10% Triton X-100 in D_2O and sonicating the sample for 15 minutes), the PDMS signal from the release of the SiGHT bound to cells appears.

Animals

A total of 23 8-week-old BALB/c mice were included in this study. Nine mice underwent a study of the biocompatibility of POEGA-b-PDMS. The remaining 14 mice underwent MRI/FLI experiments, all

receiving a 1:1 (v/v) mixture of fluorescently labeled SiGHT (50 mg mL^{-1}) and non-labeled SiGHT (50 mg mL^{-1}, to adjust the fluorescence intensity for in vivo imaging) intravenously in a total volume of 100 µL. Of these, eight were inoculated with 4T1 cancer cells (~2.5 × 10^6 cells in 100 µL PBS per mouse) by subcutaneous injection into the right flank. Tumors were allowed to grow for approximately two weeks until they reached a sufficient size for imaging (∼50-100 mm^3). The remaining six mice served as tumor-free controls. All animals subjected to imaging experiments were sacrificed for ex vivo biodistribution analysis. For imaging experiments, general anesthesia was induced with 5% isoflurane (Baxter, Deerfield, USA) and maintained at 1.5–0.5% during imaging. To prevent corneal damage, an eye gel (Ophtalmo-Septonex, Zentiva, Czech Republic) was applied prior to imaging. For MRI measurements, respiratory rate was continuously monitored using a Small Animal Monitoring System equipped with a respiratory pneumatic pillow sensor (SA Instruments, New York, USA), positioned beneath the abdomen. A heating pad was used to maintain body temperature throughout the scanning period. Specific imaging time points are detailed in the corresponding sections. Animals were housed under a 12-hour light/dark cycle at a room temperature of 22 ± 2 °C and a relative humidity of 50%. Mice were provided with a standard chow diet and had ad libitum access to tap water. To ensure good welfare and minimize stress throughout the experiments, mice had access to a variety of enrichment items, including nesting material and shelters. Mice were monitored daily. All animal protocols were approved by the Ethics Committee of the Institute for Clinical and Experimental Medicine and the Ministry of Health of the Czech Republic (No. 58/2014) in accordance with the European Communities Council Directive (2010/ 63/EU).

Dose tolerability study

To further evaluate biocompatibility, nine healthy female BALB/c mice, at eight weeks of age, were assigned to a dose tolerability study, with three receiving PBS, three administered a low-dose POEGA-*b*-PDMS concentration of 10 mg mL^{-1}, and three given a high-dose POEGA-*b*-PDMS concentration of 50 mg mL^{-1}, which corresponds to the concentration administered for in vivo MRI/FLI studies. Substances were intravenously delivered via the retro-orbital sinus at a volume of 100 µL. Baseline body weight was monitored for two weeks prior to administration, followed by regular weight measurements over a six-week period to assess potential systemic effects. Animals were selected randomly into groups.

Optical fluorescence imaging, Biodistribution, and Quantitation

An AMI HT optical imaging system (Spectral Instruments Imaging, USA) was used for both in vivo and ex vivo fluorescence imaging to assess the biodistribution of the POEGA-SCy7.5-*b*-PDMS tracer. The measurements were performed using the following parameters: excitation filter = 745 nm; emission filter = 790 nm; exposure time = 1 s, binning = 2. For in vivo data acquisition, subjects were imaged in two

orientations, first in the supine position, in which the tumor was concealed, and then in the side-lying position, where the tumor was exposed. Prior to imaging, the chest, abdomen, and tumor region were shaved to reduce light absorption and scattering, thereby enhancing fluorescence signal sensitivity. The in vivo distribution of the developed tracer was evaluated in mice, including both a negative control group and a subcutaneous tumor-bearing group. Tumor-bearing mice were randomly assigned into two groups according to sacrifice time points: day 2 (n = 3), day 8 (n = 3). Tumor-free control mice were sacrificed on day 2 (n = 3) and day 8 (n = 3). The POEGA-*co*-SCy7.5Am-b-PDMS tracer was administered intravenously via the retro-orbital sinus (V = 100 μL, c = 50 mg mL^{-1}), as a reliable route for systemic delivery. Fluorescence imaging was performed at multiple post-injection (PI) time points (5 min, 4 h, 24 h, 48 h, 96 h, and 168 h). Following the in vivo imaging protocol, vital organs, including the liver, kidneys, spleen, lungs, heart, and intestines, were extracted and assessed for tracer accumulation. In tumor-bearing mice, excised tumors were also included in the analysis. Both in vivo and ex vivo data were quantified using the software analysis tool Aura (Spectral Instruments Imaging, USA). The appropriate ROIs surrounding the tissues were selected with the Freehand tool to assess the total emission (photons/second). Ex vivo total emission values were normalized to the tissue area (photons/second/mm^2) to minimize systematic errors and improve the accuracy of the quantitative analysis. Unless otherwise specified, all statistical data are presented as mean ± standard deviation.

Histological Examination: To verify the non-toxicity and biocompatibility of the tracer, the standard histological protocol with hematoxylin-eosin (H&E) staining to detect any potential pathologies on euthanized mouse 8 days after intravenous injection of the studied silicone nanoparticles (V = 100 μL, c_{pol} = 50 mg mL^{-1}) was conducted.

Magnetic resonance imaging and spectroscopy

MRI experiments were performed using a preclinical 7T MR scanner (Bruker Biospec 70/30, Ettlingen, Germany). MR imaging of the developed metal-free organosilicon compounds was achieved using a spectrally resolved ^{1}H CSI sequence, enabling the frequency-selective visualization of the tracer. To characterize tracer sensitivity, we performed in vitro measurements in Eppendorf tube phantoms, utilizing a volume coil for signal transmission and a 4-channel 2×2 surface array coil for signal receiving. This setup allowed multiple phantom tube placements. Comparisons of the sensitivity of PEG-TMS, PEG-SIS, emulsified PDMS and POEGA-*b*-PDMS among two series of samples diluted with distilled water (a series of equivalent mass concentrations c = 5 mg mL^{-1} and second series of equivalent concentrations of traceable hydrogens c = 132 mM; PEG-SIS and PEG-TMS samples were corrected for functionalization) were performed using a ^{1}H CSI sequence with the following parameters: echo time (TE) = 10 ms, repetition time

= 1200 ms, field of view (FOV) = 25×25×10 mm^3, matrix = 32×32, and scan time = 9 min. The cellular uptake of POEGA-*b*-PDMS was confirmed via ^{1}H CSI with the following parameters: TE = 17.8 ms, TR = 1200 ms, FOV = 25×25×3.75 mm^3, matrix = 32×32, and scan time = 1 hour. The T_2 relaxation time of POEGA-*b*-PDMS was determined by analyzing signal decay at 0 ppm across a series of prolonging echo times, with the following parameters for ^{1}H CSI: echo time (TE) = 16.5–1000 ms, repetition time (TR) = 2000 ms, field of view (FOV) = 20×20×4 mm^3, matrix = 32×32, and scan time = 2 hours per scan as well as for ^{1}H PRESS: TE = 16.5–2000 ms, TR = 4000 ms, FOV = 4×6×4 mm^3, scan time = 30 minutes/scan. POEGA-*b*-PDMS sensitivity was assessed at concentrations ranging from 5 to 0.31 mg mL$^{-1,}$ with TE = 16.5 ms, TR = 850 ms, FOV = 20×20×4 mm^3, matrix = 32×32, and scan times ranging from 3.5 to 120 minutes.

For in vivo experiments, a quadrature transmit-receive cryo-probe was used to ensure optimal performance. The acquisition parameters for ^{1}H CSI were set as follows: TE = 22 ms, TR = 1200 ms, FOV = 22×22 mm^2, matrix = 16×16, and scan time = 20 min. The water suppression (VAPOR technique) bandwidth was set at 2000 Hz (~6.6 ppm) to eliminate the dominant water peak while also suppressing unwanted metabolite signals. Fat suppression was applied at a frequency offset of -3.5 ppm relative to water, with a bandwidth of 600 Hz (~2.0 ppm), effectively reducing lipid signal contamination. The working frequency was shifted to -1.5 ppm, and the spectral width was set to 5.1 ppm. The shimming area was adjusted individually to optimize field homogeneity. The ex vivo ^{1}H CSI parameters were as follows: TE = 40 ms, TR = 1200 ms, FOV = 72×72 mm^2, and matrix = 48×48. Reference ^{1}H MR images were acquired using a standard T_2-weighted fast spin–echo with the following parameters: TE = 33 ms, TR = 2500 ms, rare factor = 8, matrix = 256×256, and FOV = 22×22 mm^2, for reference ex vivo imaging, the parameters were as follows: TE = 45 ms, TR = 2200 ms, rare factor = 8, and matrix = 512×512, and FOV = 72×72 mm^2. The potential clinical translatability of POEGA-*b*-PDMS was assessed on the clinical 3T MR scanner VIDA (Siemens Healthineers, Erlangen, Germany) by measuring the signal intensity of a 50 mL Falcon tube filled with SiGHT (20 mg mL^{-1}) placed next to the head of a volunteer. MRI measurements involving a human volunteer were performed with written informed consent; the phantom was positioned adjacent to the subject solely for imaging reference. We utilized a body transmit coil with a volume head coil for signal receiving. Anatomical localization was performed using a standard turbo spin echo sequence (TR/TE = 5000/89 ms, 4 mm slice thickness). To acquire spatially localized information about POEGA-*b*-PDMS, ^{1}H CSI was used (TR/TE = 1700/30 ms, matrix = 16×16, scan time = 8 min).

MRI data processing and quantitation

Baseline correction was applied to in vivo and ex vivo ^{1}H CSI data via a built-in module for spectroscopy provided in Paravision 360 software (V3.6; Bruker, Ettlingen, Germany). The 'cyan-hot'

colormap was applied in postprocessing to bilinearly interpolated ^{1}H CSI data, enabling the co-registered visualization of tracer distribution with the reference ^{1}H MRI. Signal decay for T_2 evaluation and the concentration sensitivity of the tracers were assessed by calculating the signal-to-noise ratio (SNR), determined within a defined region of interest (ROI) by dividing the mean signal intensity (S) by the average standard deviation of noise (σ) measured in the void regions. To account for the intrinsic Rician noise distribution inherent in MR images, the SNR values were adjusted by a factor of 0.655. The data from the clinical 3T MR system VIDA were processed using 3rd-order polynomial baseline subtraction, followed by integration with a custom script (supplementary information).

Statistical analysis

Unless otherwise stated, all values are given as the mean ± standard deviation (SD). Statistical analysis was performed using GraphPad Prism (GraphPad Software, Boston, Massachusetts, USA). Statistical significance was determined by a two-tailed unpaired Student's t test with Holm–Sidak correction: ns, not significant; *p < 0.05; **p < 0.01; ***p < 0.001.

Post-processing R-code for a clinical 3T scanner

```
nmr<-read.csv("CSV.csv",sep=';',header=F, stringsAsFactors=FALSE)
bcg<-c(205:230)
bl<-c(200:210,225:235)
integral<-0
for (n in 1:256) {
  fit<- lm(nmr[bl,n]~poly(bl,3,raw=TRUE))
  pred<-(coef(fit)[1]+(coef(fit)[2])*bcg^1+(coef(fit)[3])*bcg^2+(coef(fit)[4])*bcg^3)
  nmrcor<-(as.numeric(nmr[bcg,n])-pred)
  int<-sum(nmrcor)
  integral<-c(integral,int)
 }
integral<-integral[-1]
M<-matrix(integral,nrow=16,ncol=16)
Mpos<-pmax(M,0)
write(Mpos, "data.txt")
```

Scheme S1. Photoiniterter-RAFT synthesis of PDMS-b-POEGMA block copolymer (SiGHT)

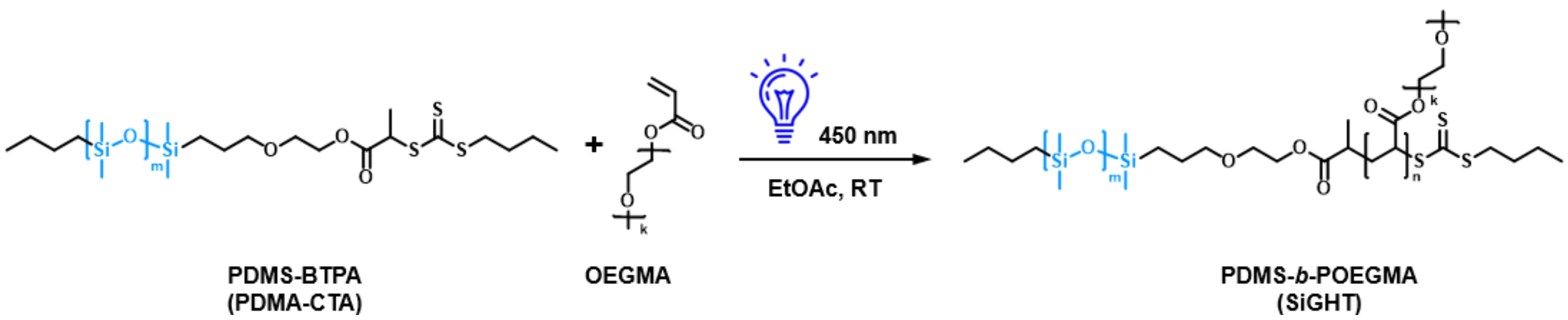

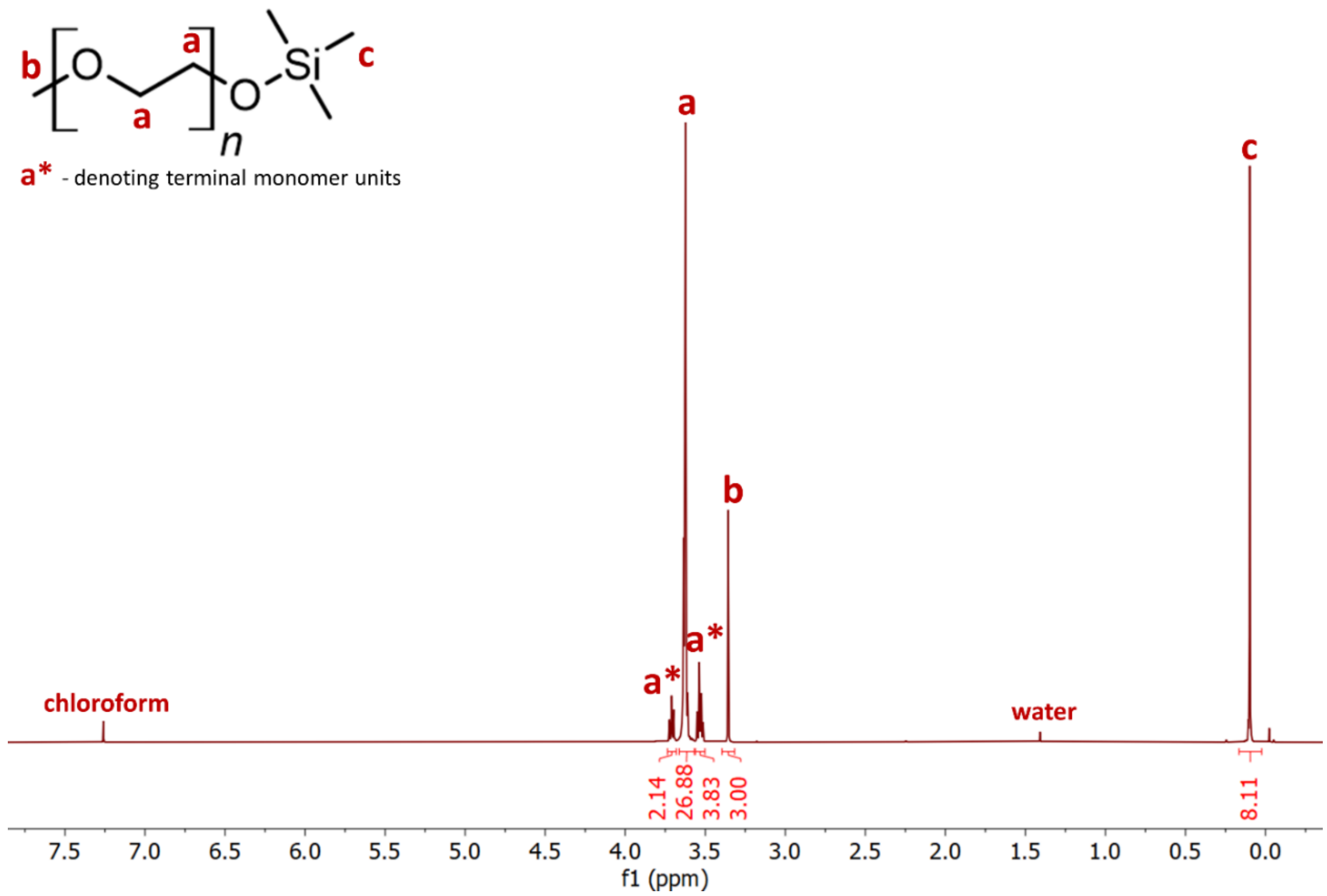

Figure S1. ^{1}H NMR of PEG-TMS in $CDCl_3$.

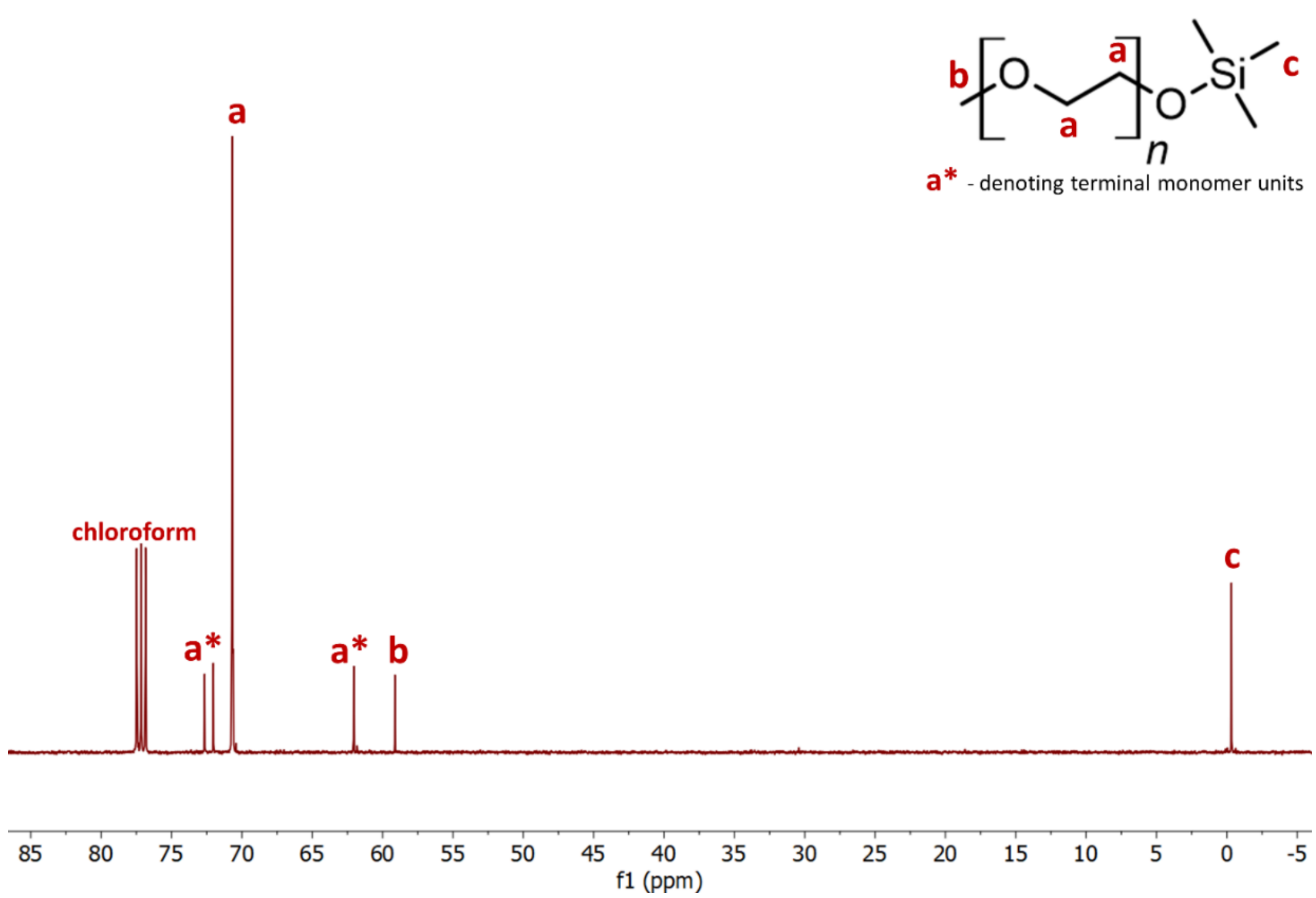

Figure S2. ^{13}C NMR of PEG-TMS in $CDCl_3$.

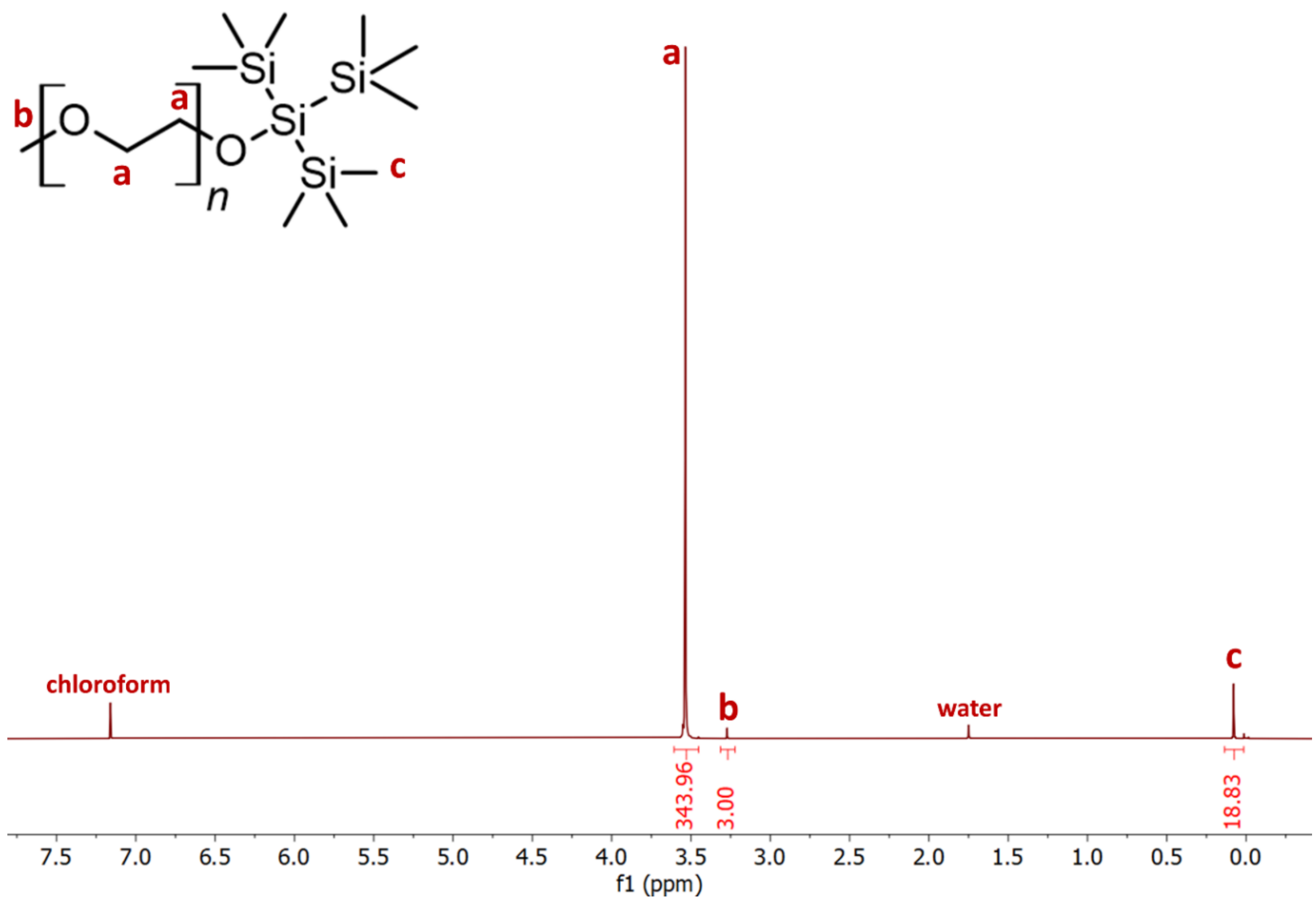

Figure S3. ^{1}H NMR of PEG-SIS in $CDCl_3$.

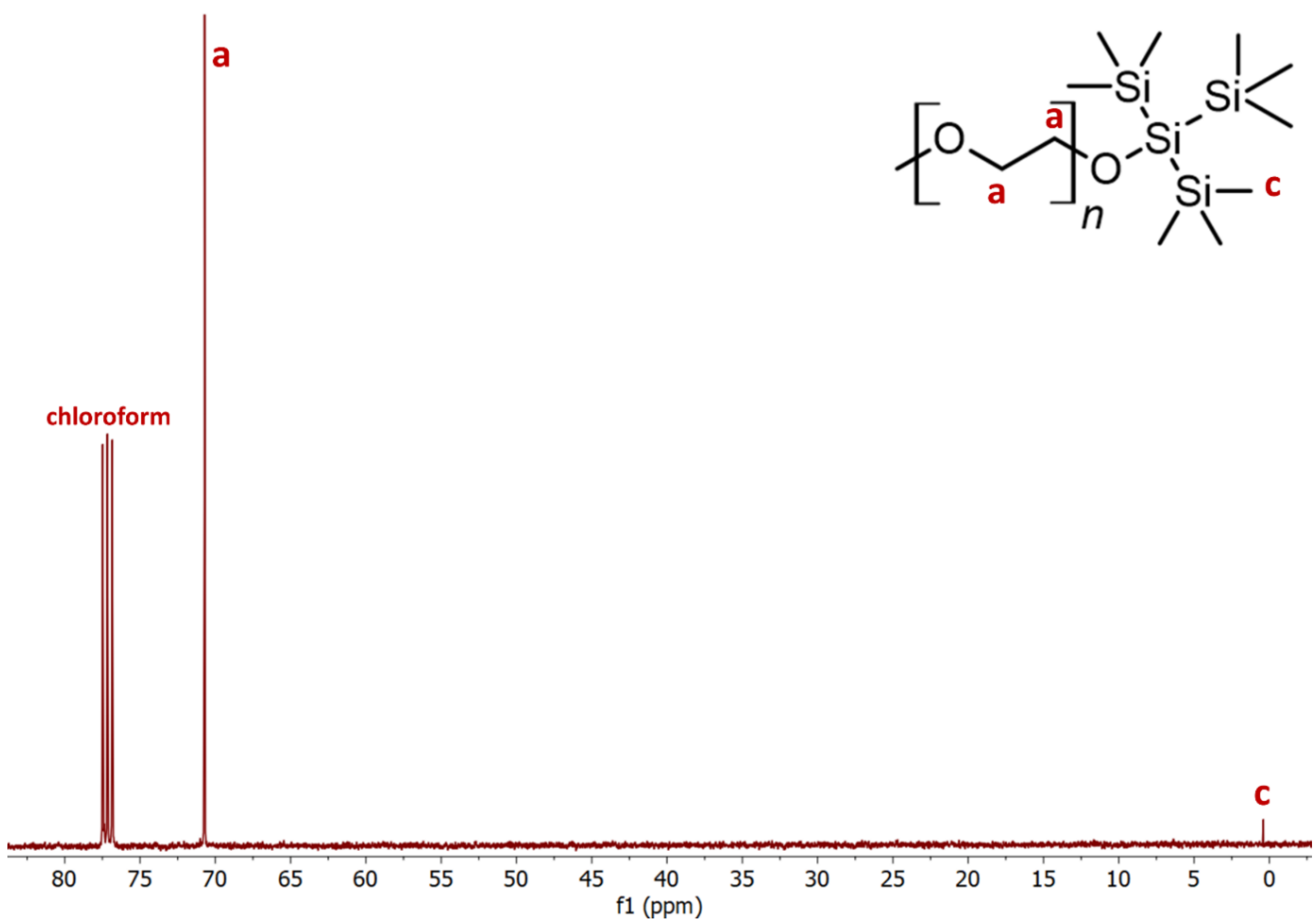

Figure S4. ^{13}C NMR of PEG-SIS in $CDCl_3$.

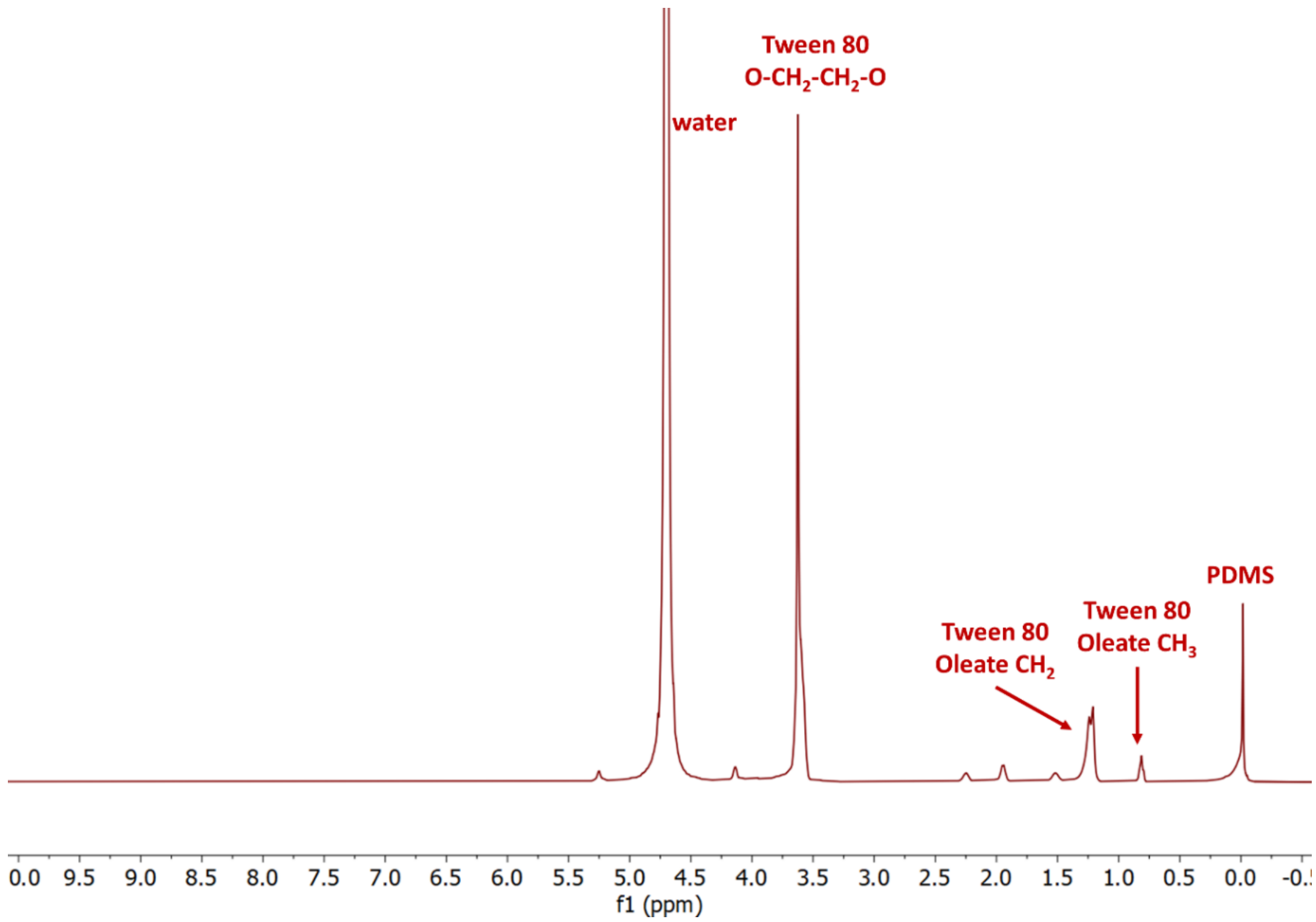

Figure S5. ^{1}H NMR of PDMS-Tween 80 emulsion in 10% H_2O/ 90% D_2O (v/v).

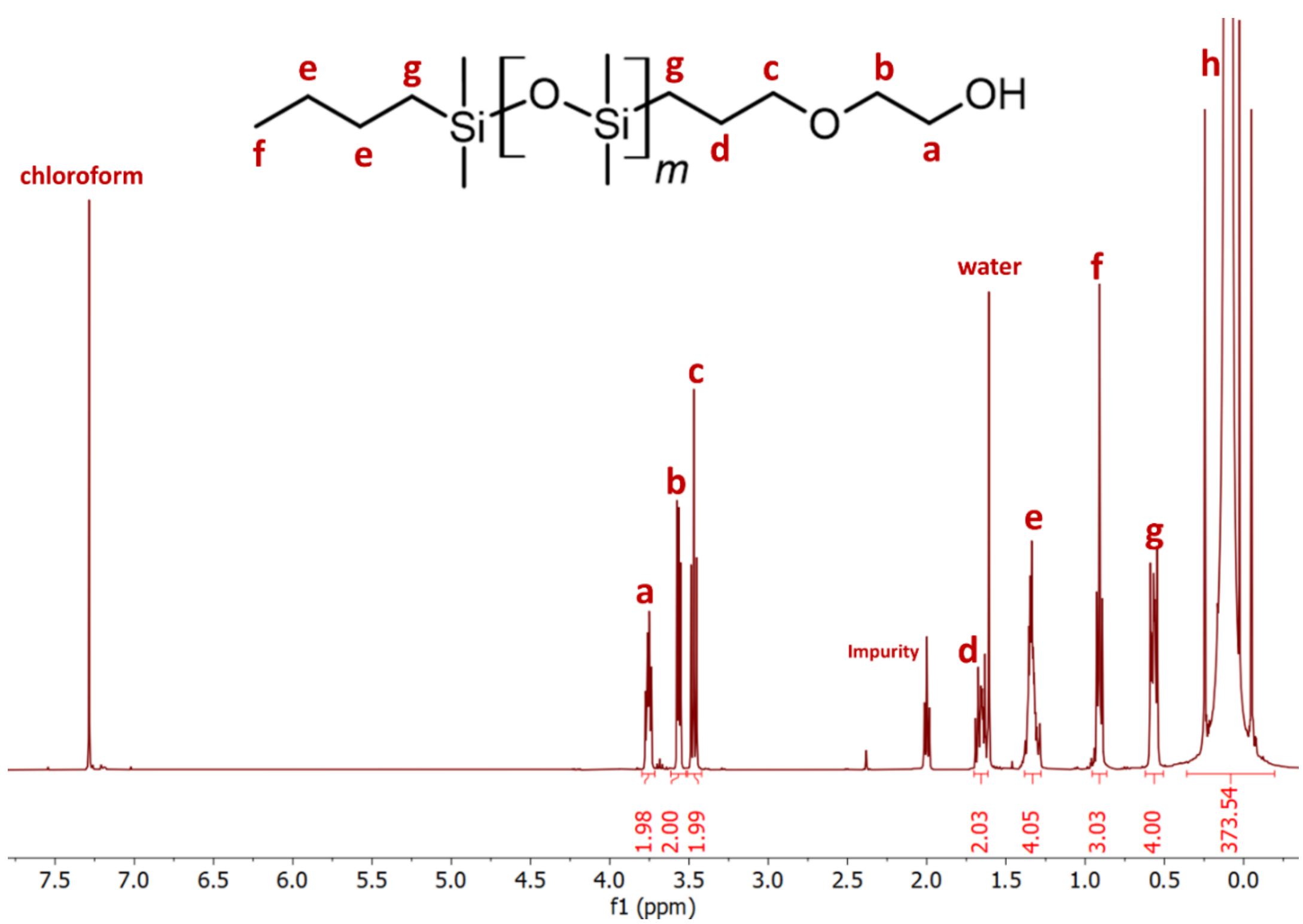

Figure S6. ^{1}H NMR of commercial monohydroxy-PDMS in $CDCl_3$.

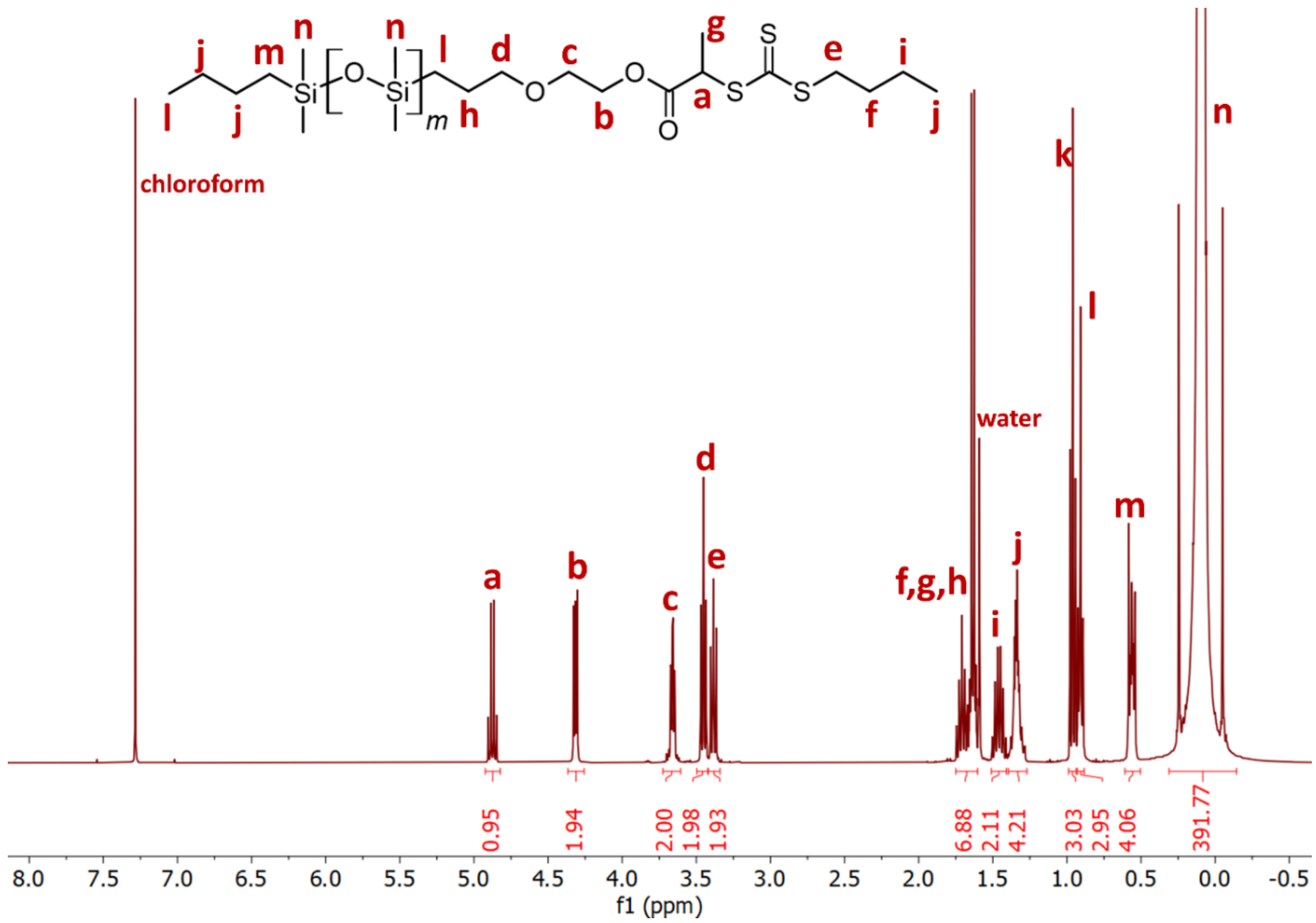

Figure S7. ^{1}H NMR of PDMS-CTA in $CDCl_3$.

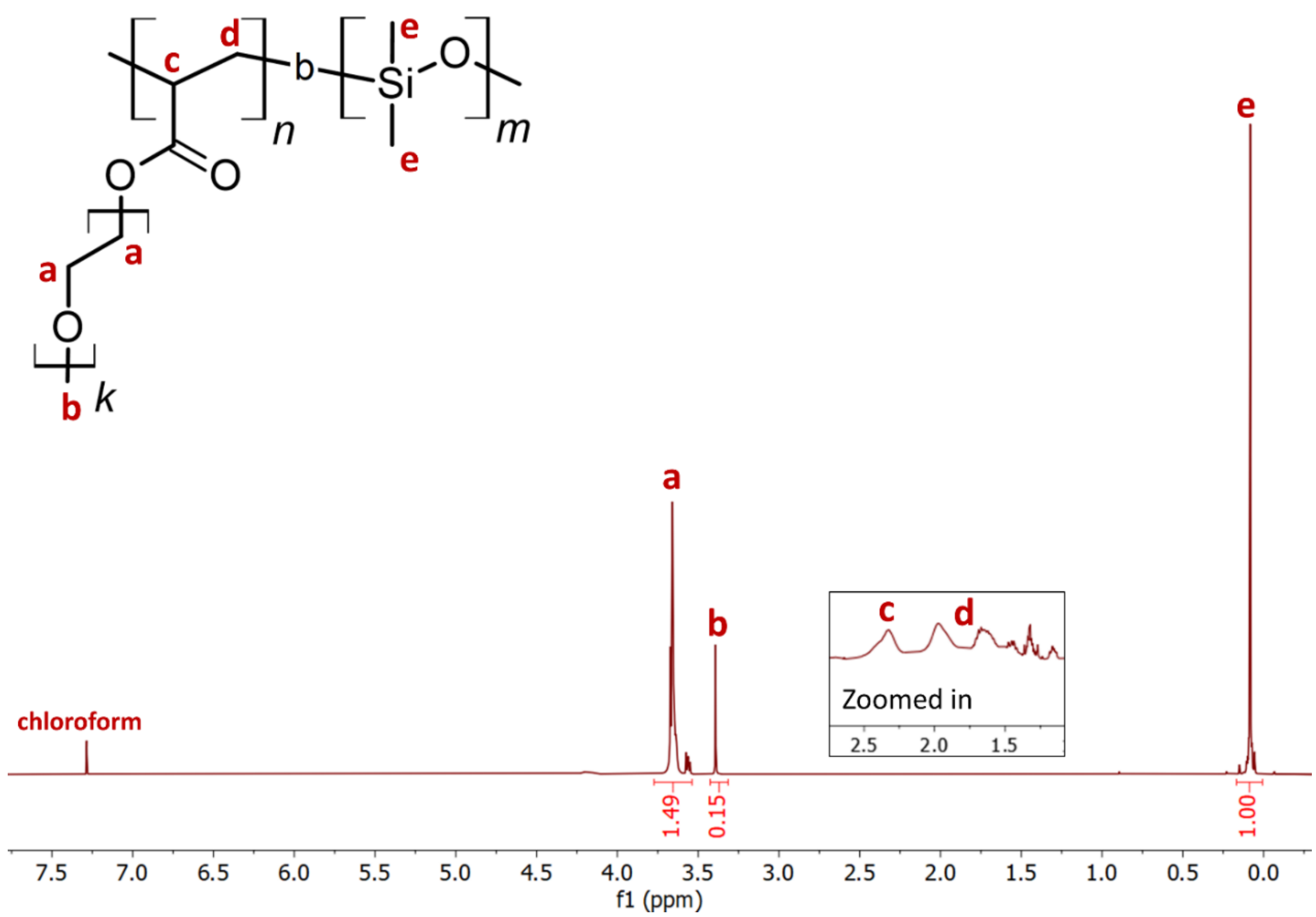

Figure S8. ^{1}H NMR of POEGA-*b*-PDMS (SiGHT) in $CDCl_3$ (polymer end groups omitted for clarity).

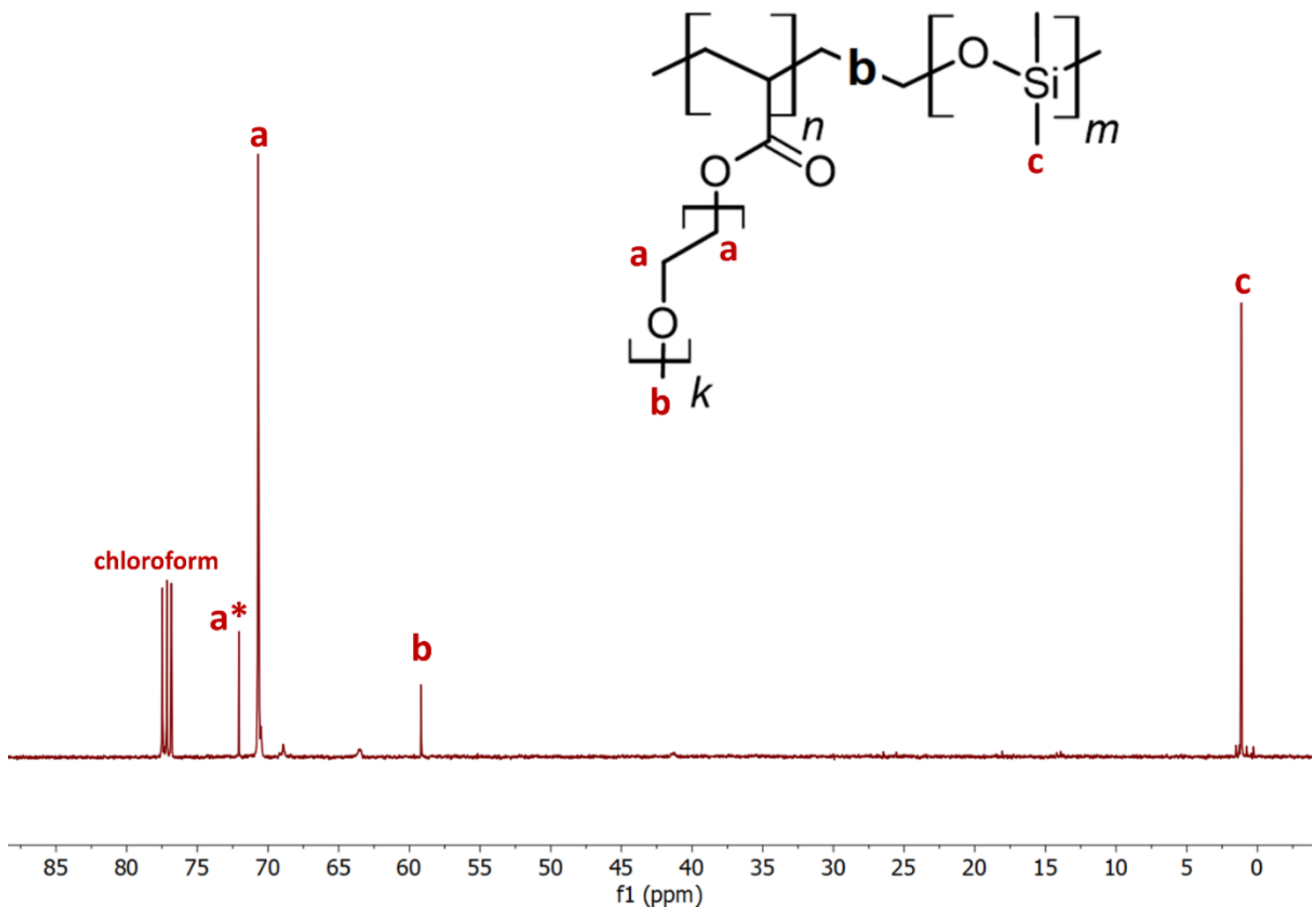

Figure S9. ^{13}C NMR of POEGA-*b*-PDMS (SiGHT) in $CDCl_3$ (polymer end groups omitted for clarity).

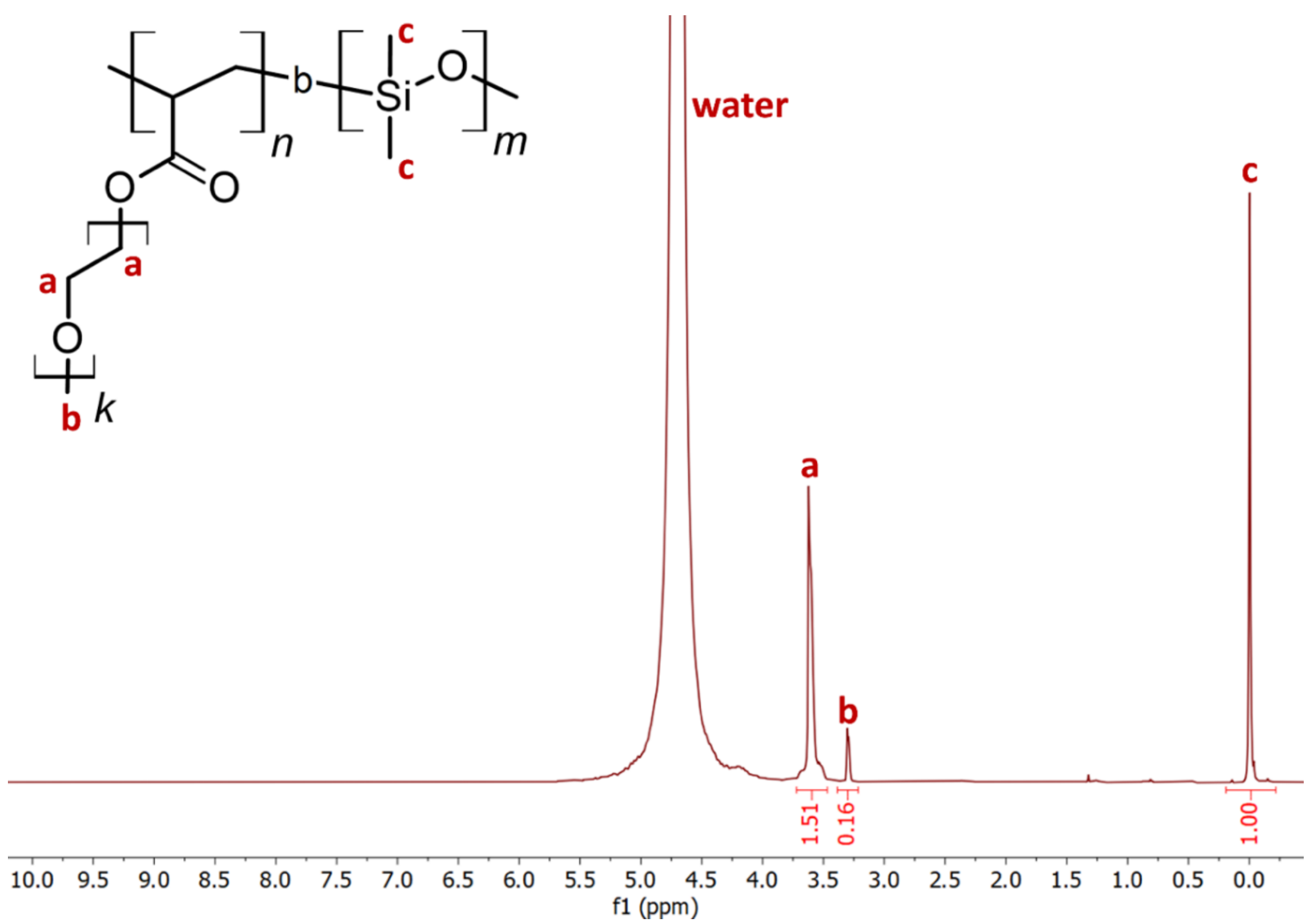

Figure S10. ^{1}H NMR of POEGA-b-PDMS (SiGHT) in 10% H_2O/ 90% D_2O (v/v) (polymer end groups omitted for clarity).

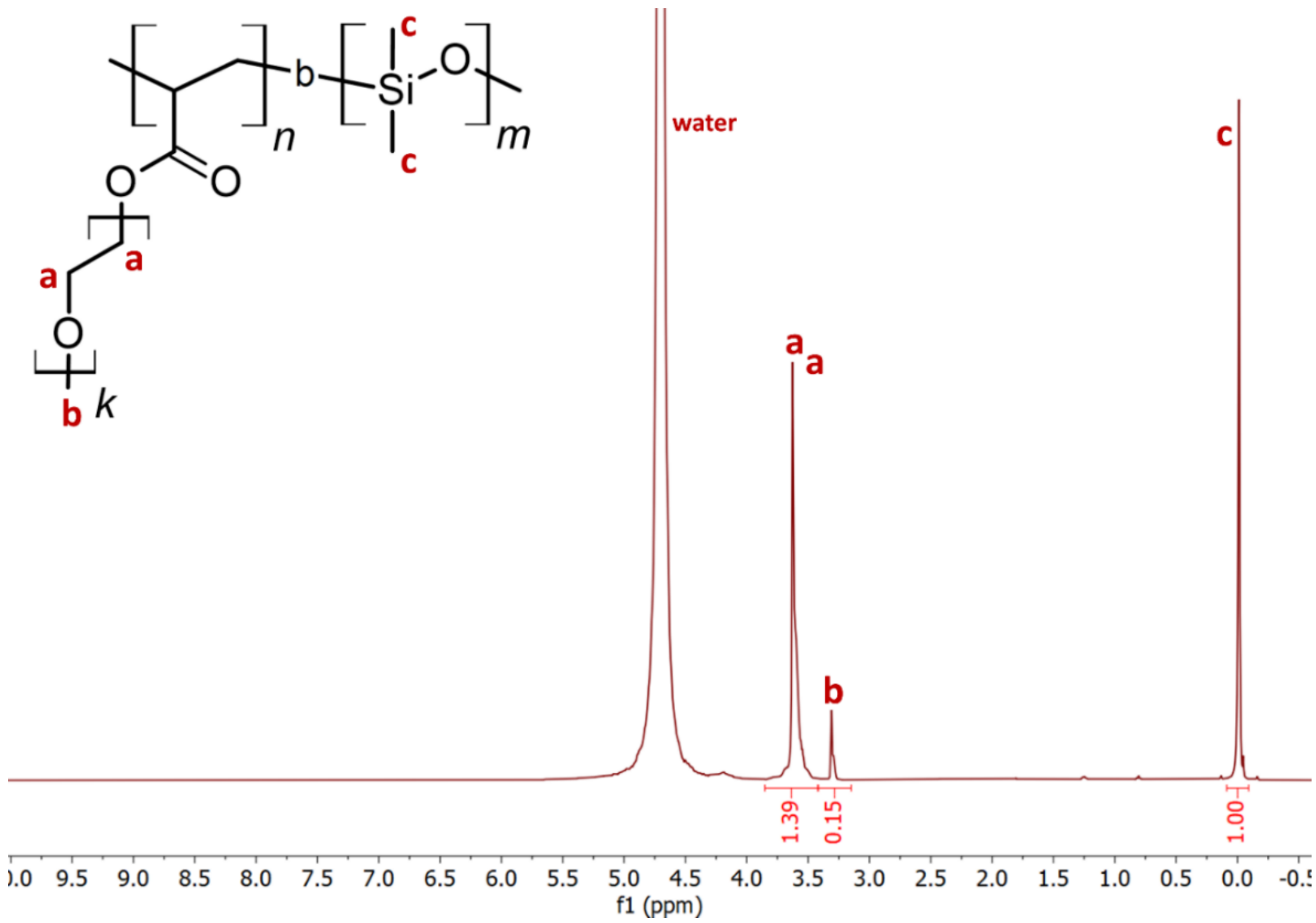

Figure S11. ^{1}H NMR of POEGA-*co*-SCy7.5Am-*b*-PDMS (fluorescently labeled SiGHT) in D_2O (polymer end groups and structure of dye omitted for clarity).

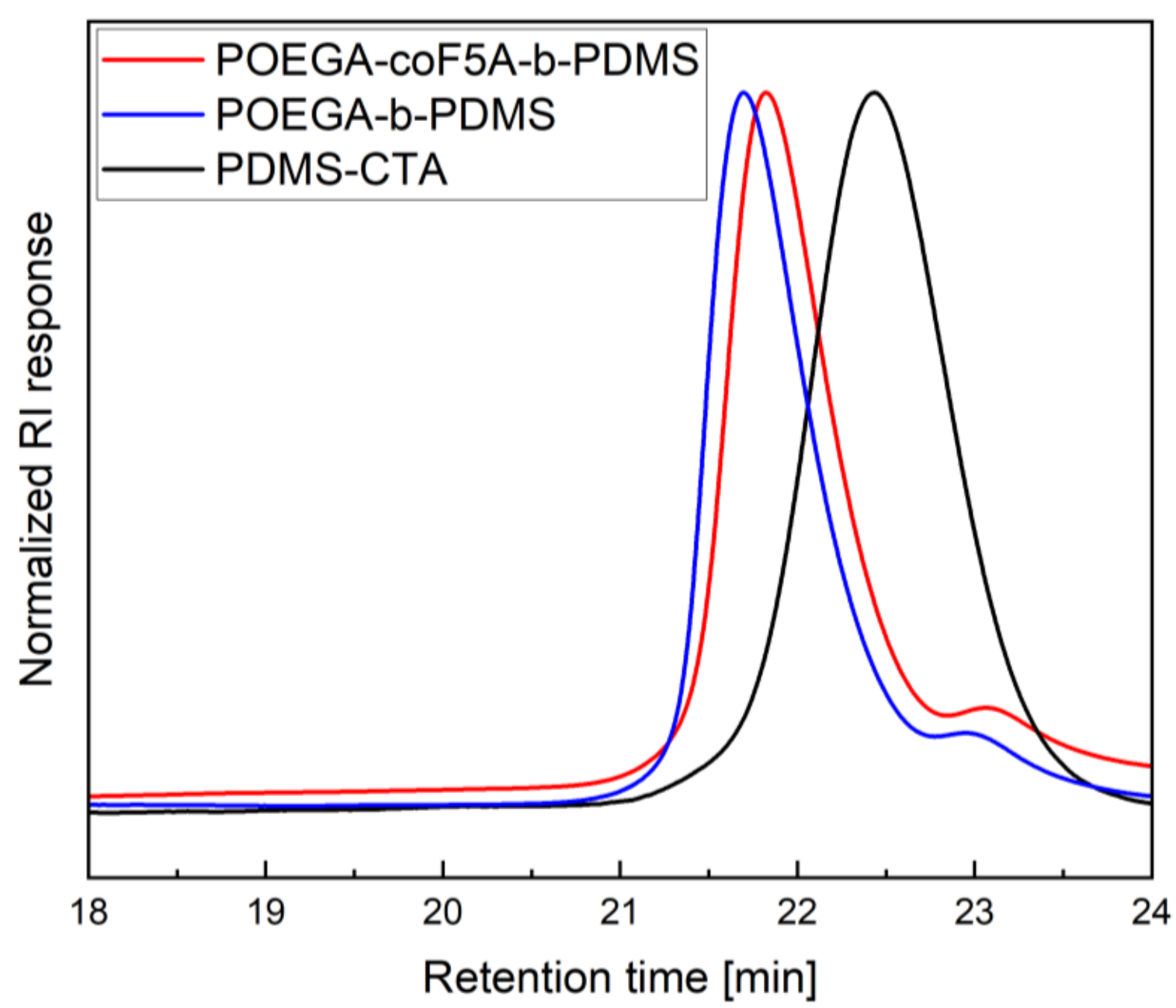

Figure S12. SEC traces of PDMS-CTA (black), POEGA-b-PDMS (red), and P(OEGA-co-(pentafluorophenyl acrylate))-b-PDMS ((PDMS-b-(POEGA-co-F5A)), blue) used for synthesis of POEGA-co-SCy7.5Am-b-PDMS, eluted with THF.

Table S1. SEC data of PDMS-CTA, POEGA-b-PDMS and P(OEGA-co-(pentafluorophenyl acrylate))-b-PDMS (PDMS-b-(POEGA-co-F5A)). M_n and $Đ$ values are based on polystyrene calibration.

Sample	M_n (kg/mol)	$Đ$
PDMS-CTA	7.9	1.07
PDMS-*b*-POEGA	10.4	1.09
PDMS-*b*-POEGA-coF5A	9.9	1.08

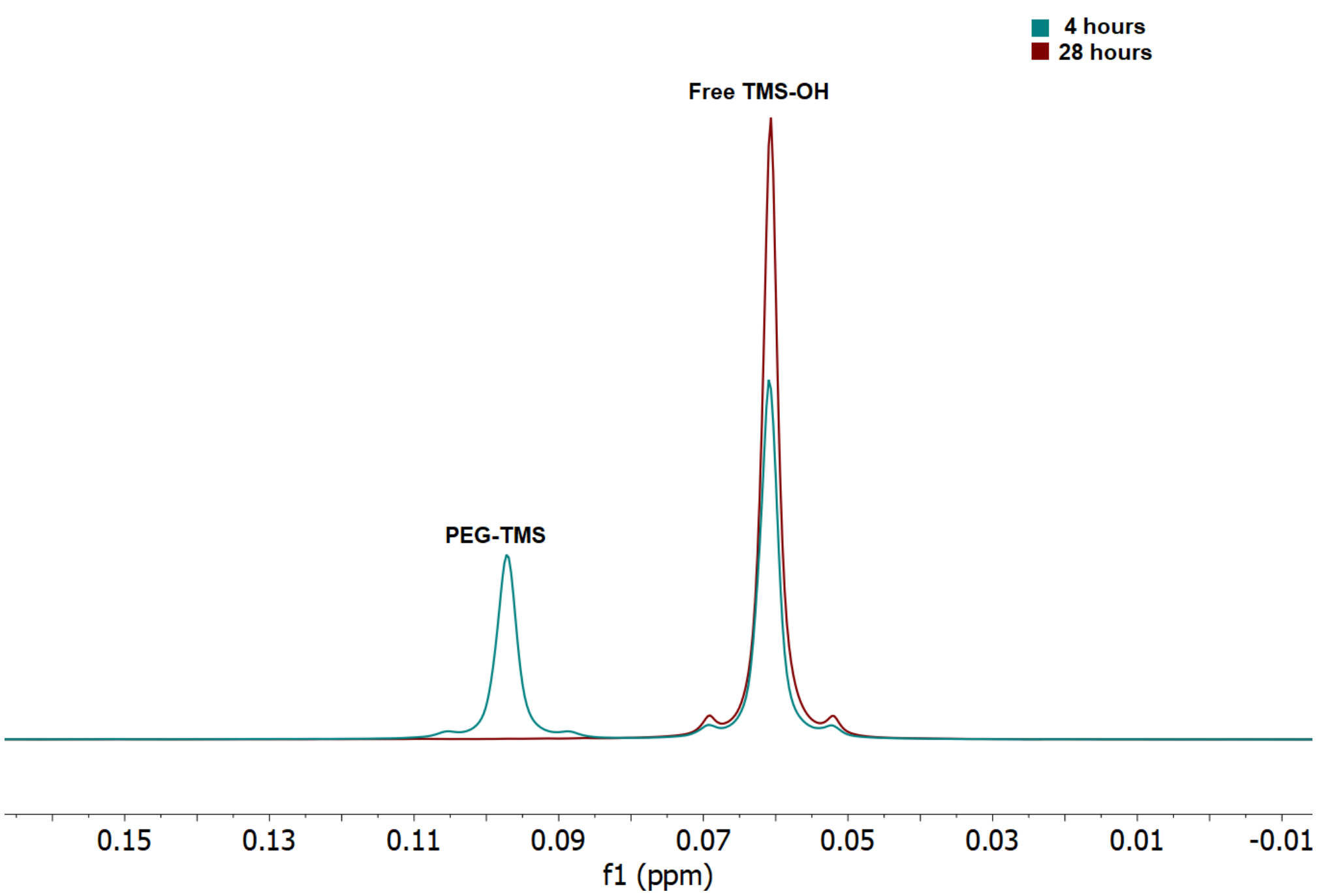

Figure S13. PEG-TMS degradation: Detail of ^{1}H NMR of PEG-TMS trimethylsilyl signal in D_2O. Blue: 4 hours after dissolution (already exhibiting notable hydrolysis), red: 28 hours after dissolution (fully hydrolyzed).

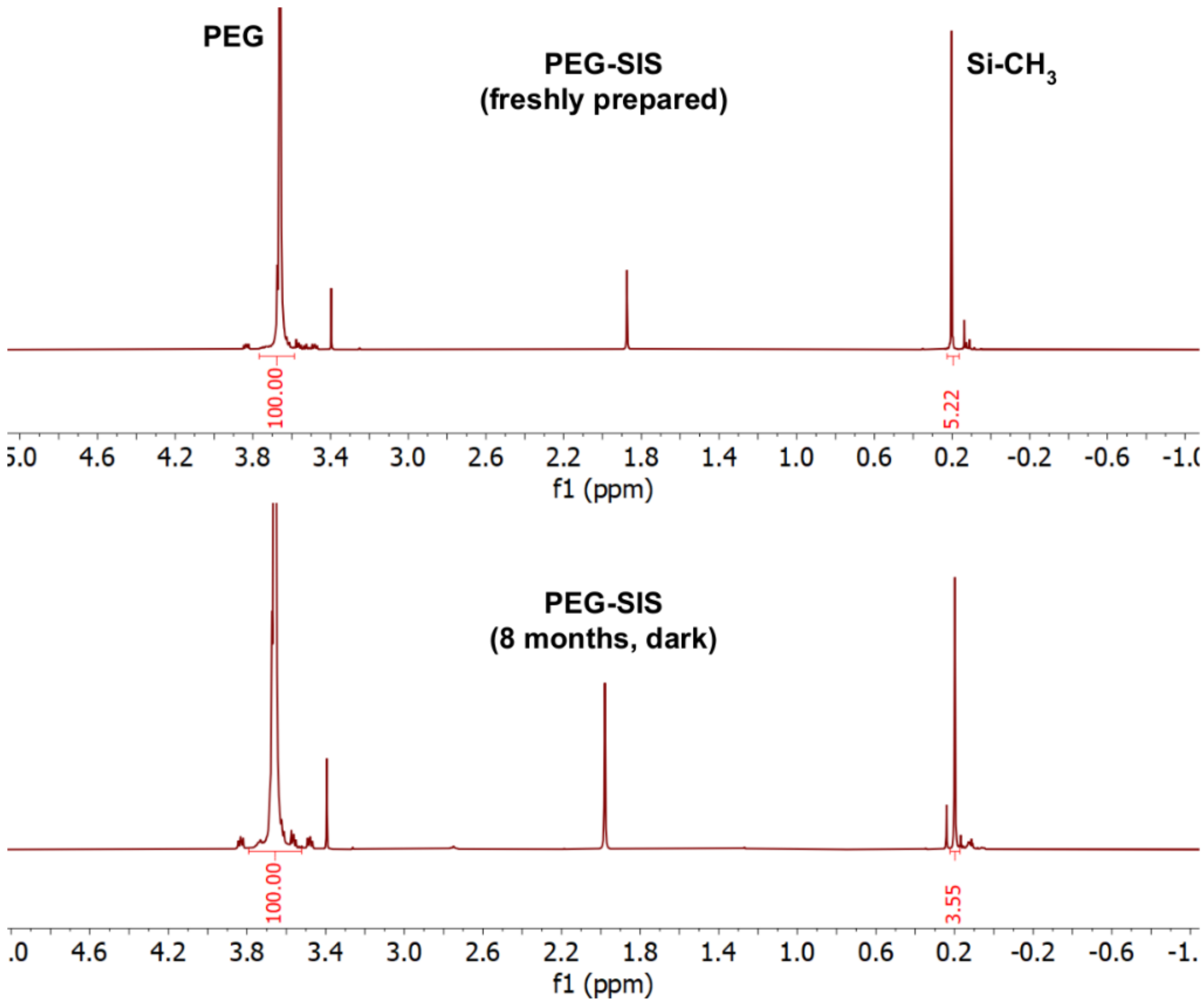

Figure S14. ^{1}H NMR spectra of PEG-SIS in $CDCl_3$ after preparation (top); after 8-month storage in dark, dry conditions, showing partial autodegradation.

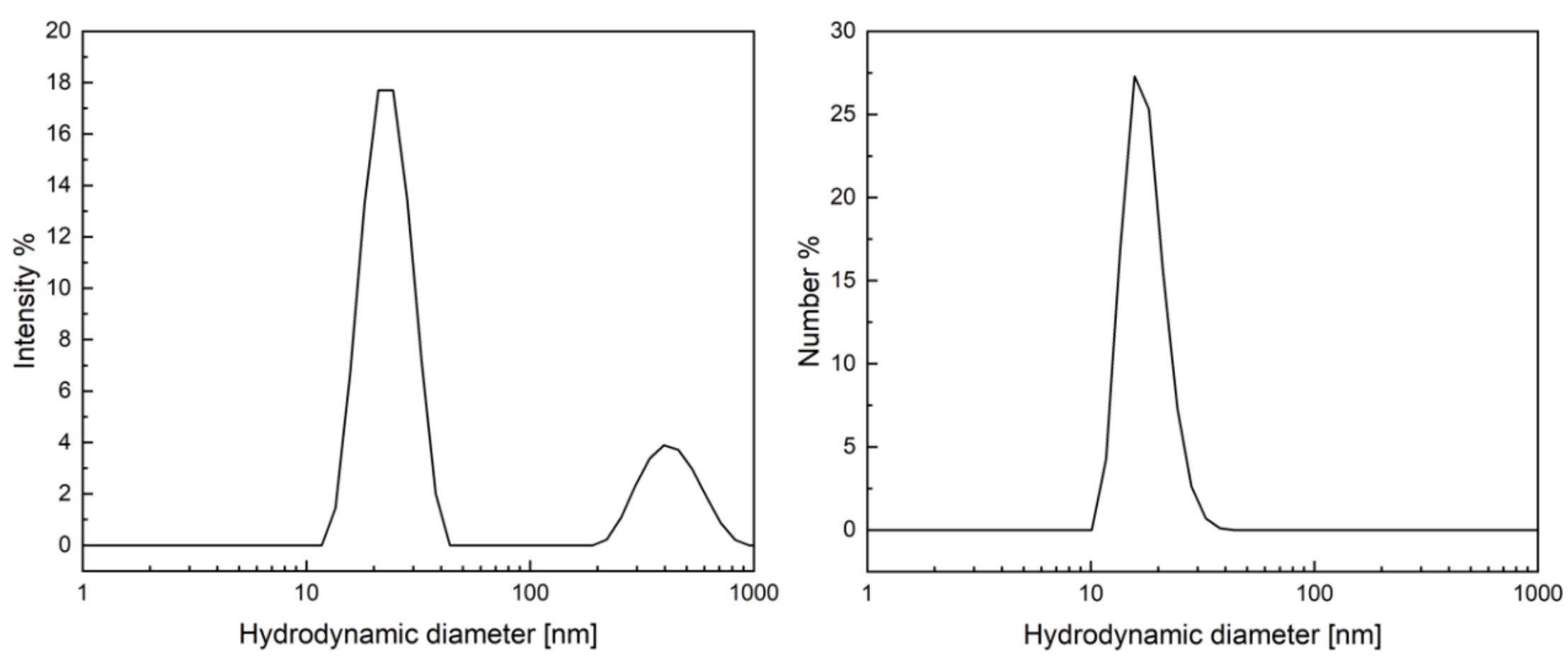

Figure S15. Dynamic light scattering analysis of hydrodynamic radius of POEGA-*b*-PDMS micelles in water (c_{pol} = 2 mg mL^{-1}, intensity- and number-weighted distributions).

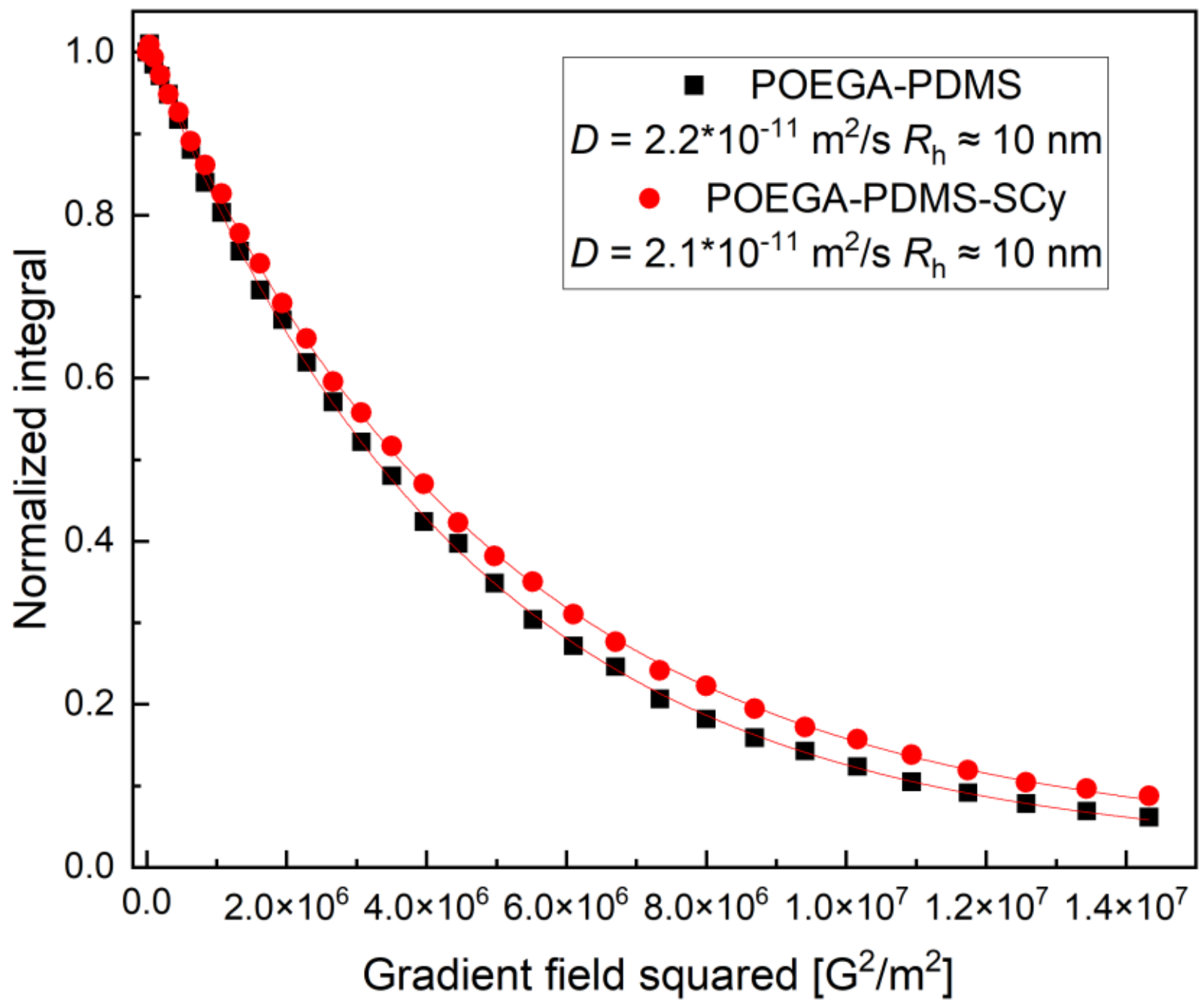

Figure S16. Dependence of the normalized integral of the PDMS signal in micelle samples of POEGA-*b*-PDMS and POEGA-co-SCy7.5Am-*b*-PDMS on gradient field strength in ^{1}H DOSY NMR experiment (measured in 90% H_2O/ 10% D_2O (v/v)), confirming almost no effect of the dye on hydrodynamic radius. The hydrodynamic radius was estimated assuming the viscosity of pure H_2O (1.0 mPa s) at a temperature of 25 °C.

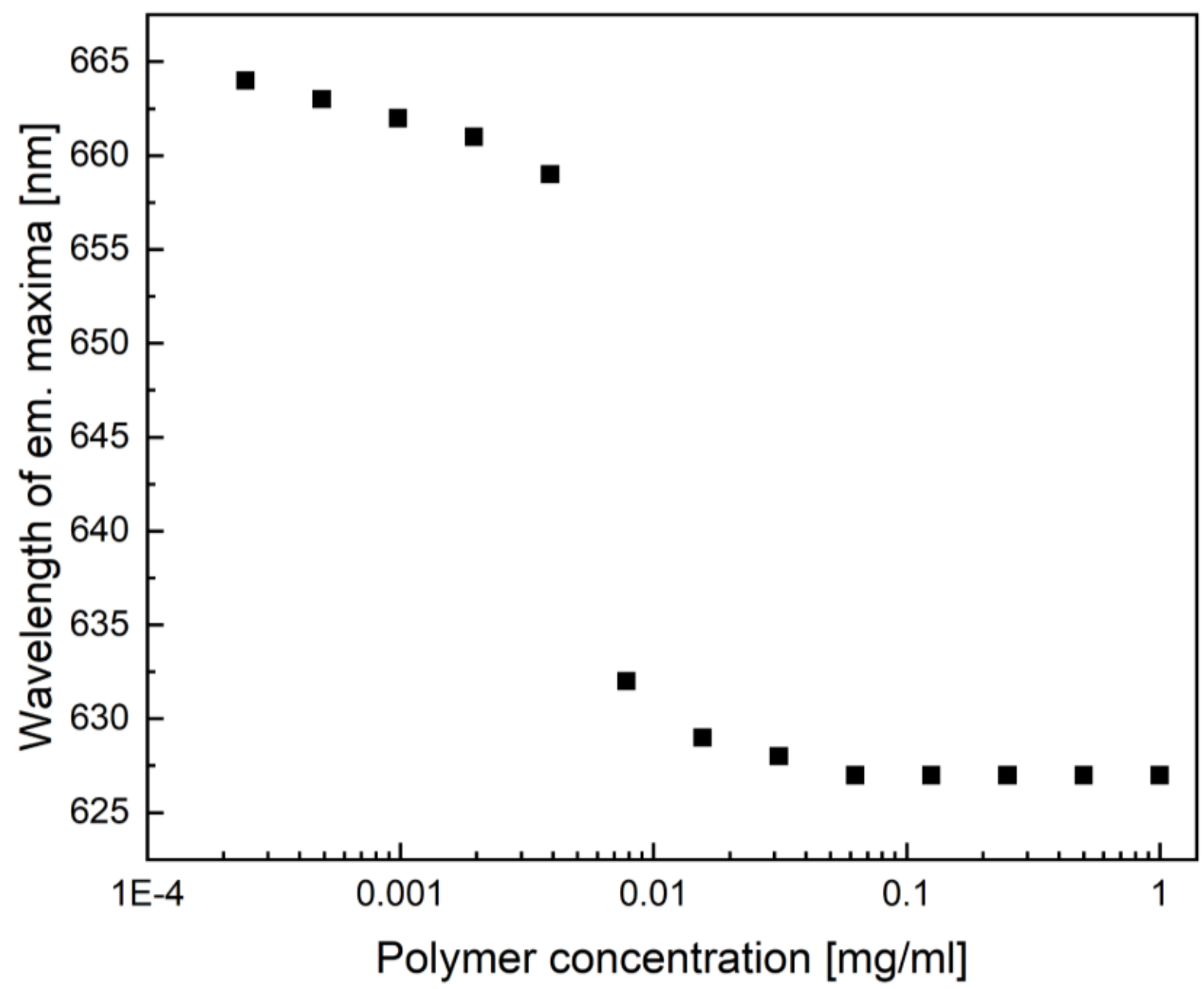

Figure S17. Determination of POEGA-b-PDMS critical micelle concentration (CMC) via fluorescence spectroscopy of Nile Red probe, dependence of emission maxima wavelength (λ_{ex} = 550 nm) on polymer concentration.

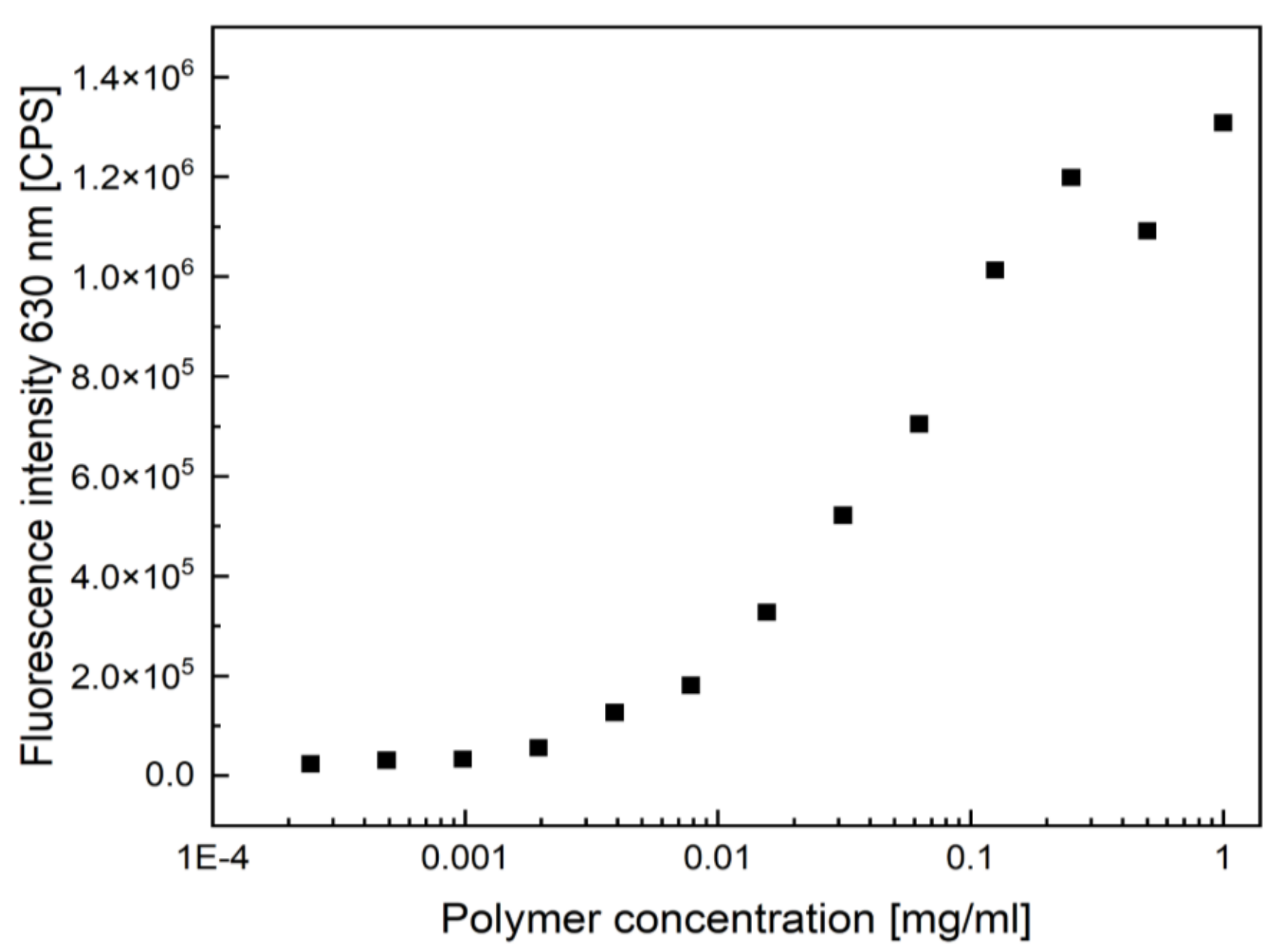

Figure S18. Determination of POEGA-b-PDMS critical micelle concentration (CMC) via fluorescence spectroscopy of Nile Red probe, dependence of emission intensity (λ_{ex} = 550 nm) at 630 nm (corresponding to probe bound to micelles) on polymer concentration.

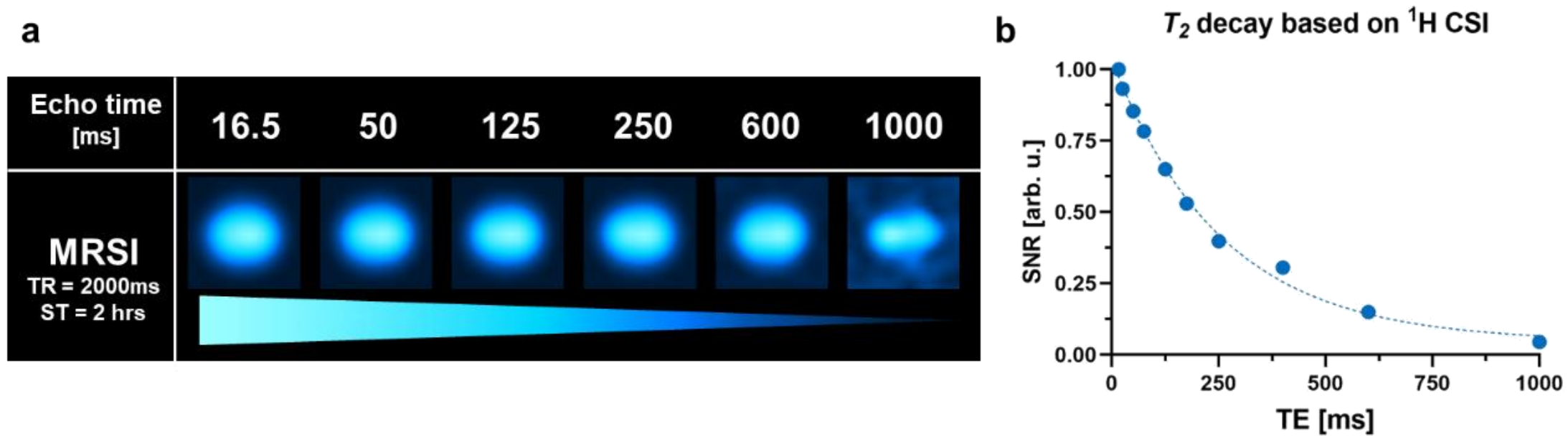

Figure S19. T_2 relaxation assessment of SiGHT using ^{1}H chemical shift imaging (CSI). **a)** ^{1}H MR signal intensity of SiGHT (tube cross-section) progressively decreases with increasing echo time. **b)** Corresponding T_2 relaxation curve derived from signal-to-noise ratio (SNR) attenuation, with one-phase decay fitting (R^2 = 0.99) yielding a T_2 value of 260 ms.

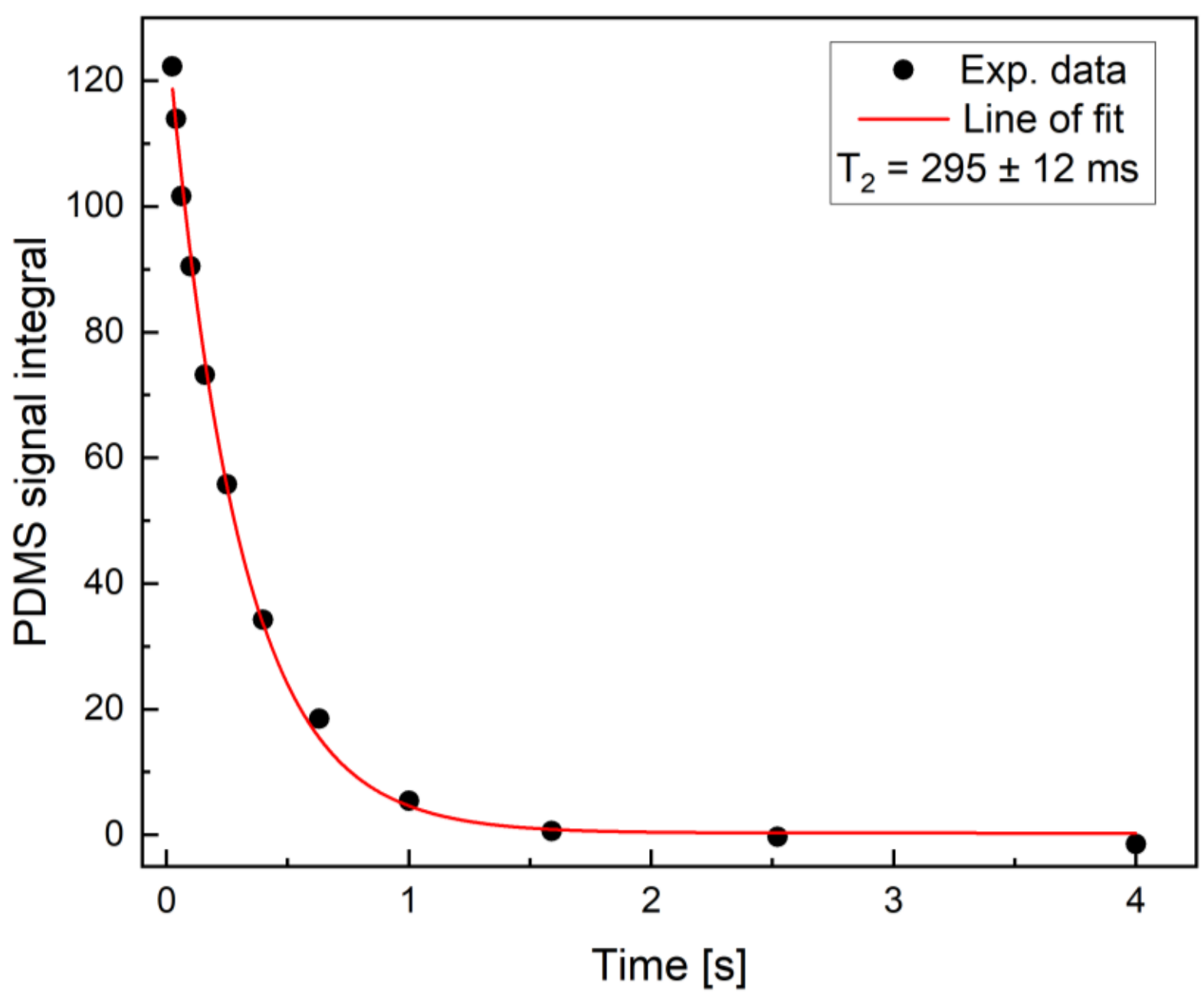

Figure S20. Measurement of T_2 (7 T, CPMG experiment) of the PDMS signal of POEGA-*b*-PDMS micelles (10% H_2O/ 90% D_2O (v/v)).

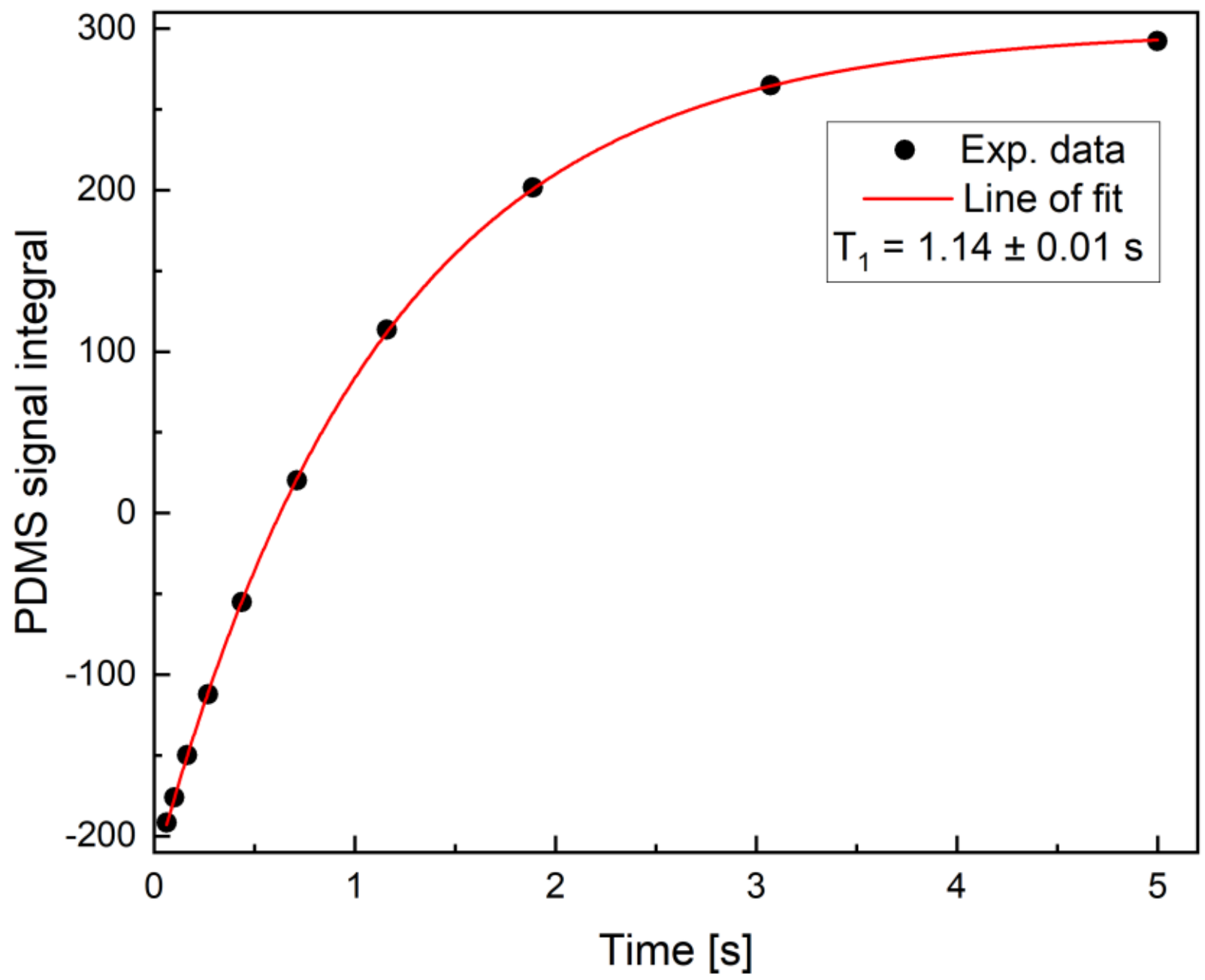

Figure S21. Measurement of T_1 (7 T, inversion-recovery experiment) of the PDMS signal of POEGA-*b*-PDMS micelles (10% H_2O/ 90% D_2O (v/v)).

Table S2. Comparison of calculated weight % of Si-CH_3 hydrogen nuclei (accounting for degree of functionalization) in prepared contrast agents (dry mass).

Sample	% (w/w)
PEG-TMS	1.6
PEG-SIS	0.4
Emulsion	1.6
SiGHT	2.7

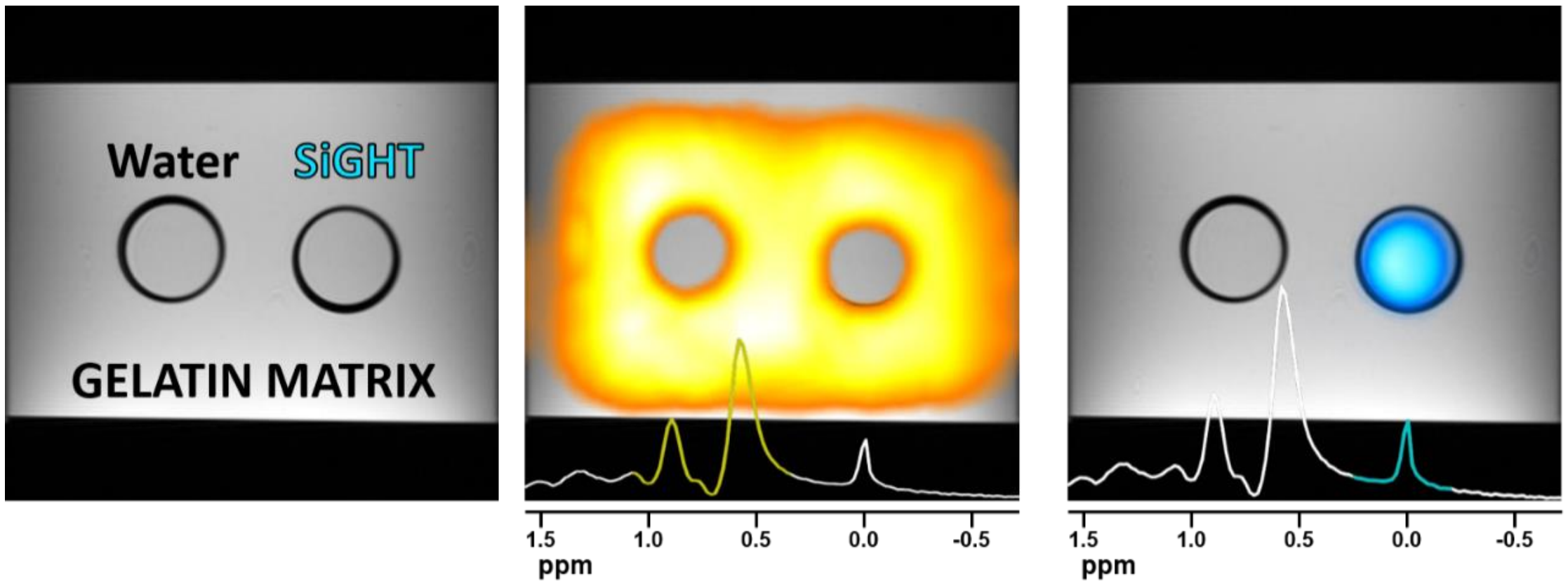

Figure S22. The tracer embedded in a porcine skin gelatin matrix to demonstrate selective visualization of either the background matrix or the SiGHT tracer without mutual interference. (left: conventional ^{1}H MR imaging, middle: overlaying ^{1}H MRI and ^{1}H CSI (~1.1–0.4 ppm spectral range), right: overlaying ^{1}H MRI and ^{1}H CSI (~0.3–-0.1 ppm spectral range).

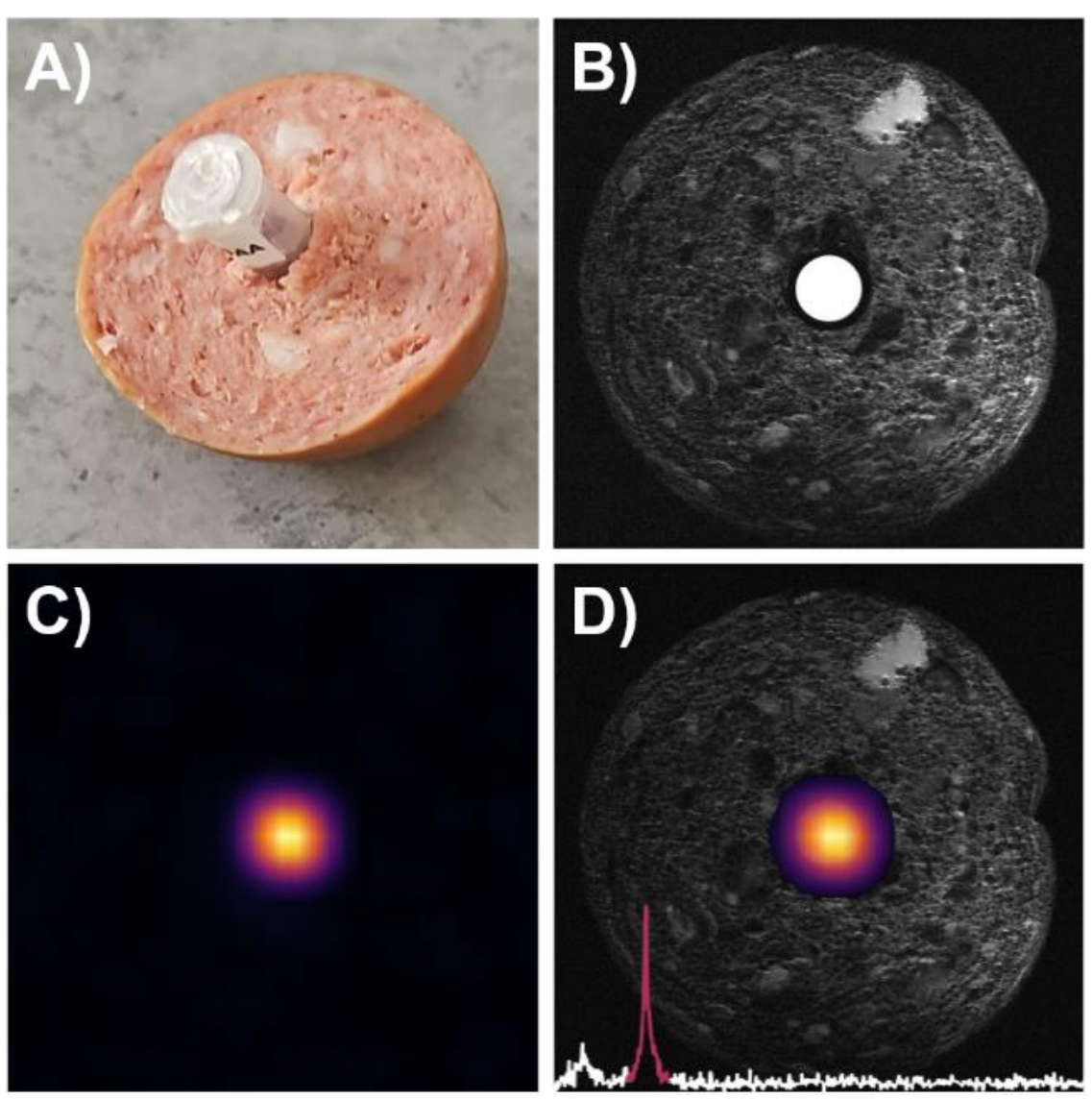

Figure S23. Validation of tracer specificity in a lipid-rich environment. (A) Photograph of the tracer tube embedded in an ex vivo porcine tissue surrogate. (B) Structural MRI of the cross-section. (C) Spectrally selective CSI map revealing the isolated organosilicon hotspot. (D) Fused anatomical and hotspot image with the corresponding spectrum, illustrating effective visualization of the tracer without interference from the completely suppressed surrounding fat.

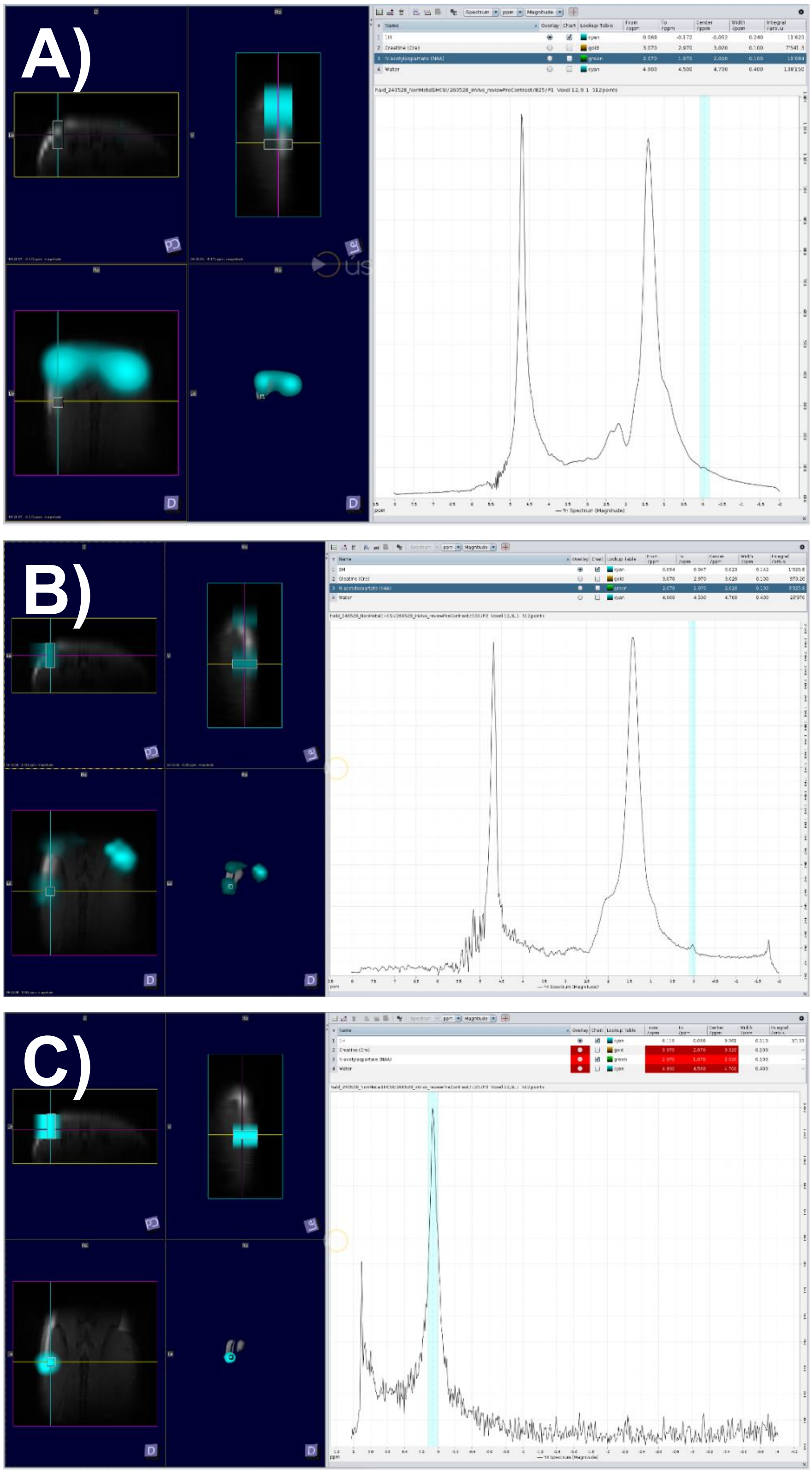

Figure S24. In vivo spectral evaluation of the SiGHT tracer and the efficacy of lipid suppression following subcutaneous administration. To assess the impact of suppression pulses on the tracer signal, data were extracted from the same selected voxel across three complementary settings, selected voxel represents the

area of subcutaneous tracer administration. (A) The full ^{1}H chemical shift spectrum (-2 to 8 ppm) acquired without fat suppression, demonstrating the massive endogenous lipid background. The tracer signal is superimposed on the lipid signal (B) The full spectral region acquired with active fat suppression. The endogenous lipid signals are substantially reduced, however we are still not able to clearly detect the tracer due to lipid artifacts (C) The targeted narrow spectral range utilized for the final in vivo hotspot imaging, acquired with fat suppression, demonstrating robust and background-free tracer detection.

The organosilicon resonance remained spectrally separable from the endogenous background and could be selectively reconstructed under the applied acquisition conditions. Although residual lipid signal was still visible after fat suppression, off-resonance lipid contributions outside the selected receiver bandwidth were removed by the digital receiver filtering and decimation pipeline before final spectral reconstruction. Therefore, no detectable lipid folding artifacts were observed in the organosilicon spectral window used for hotspot visualization.

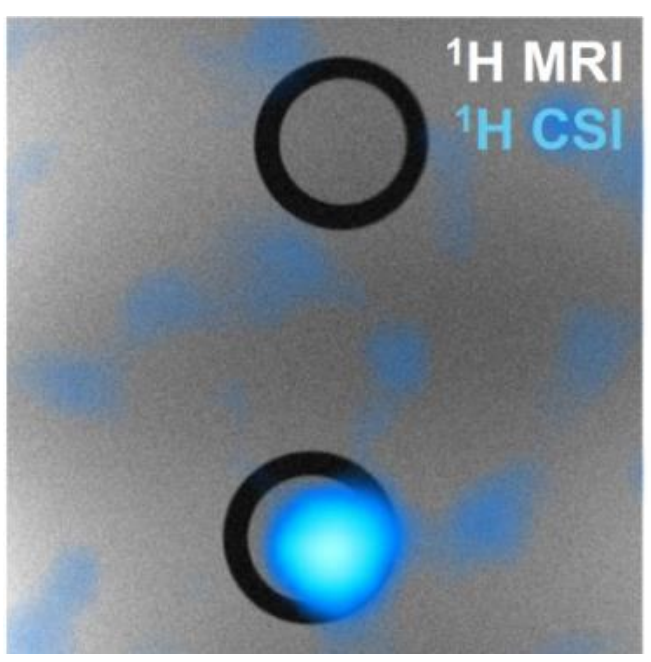

Figure S25. Verification of the presence of SiGHT SiGHT (5 mg mL^{-1}) in the lower tube by ^{1}H CSI for comparison of SiGHT (bottom tube) and PBS (top) ^{1}H MRI in **Figure 4C**.

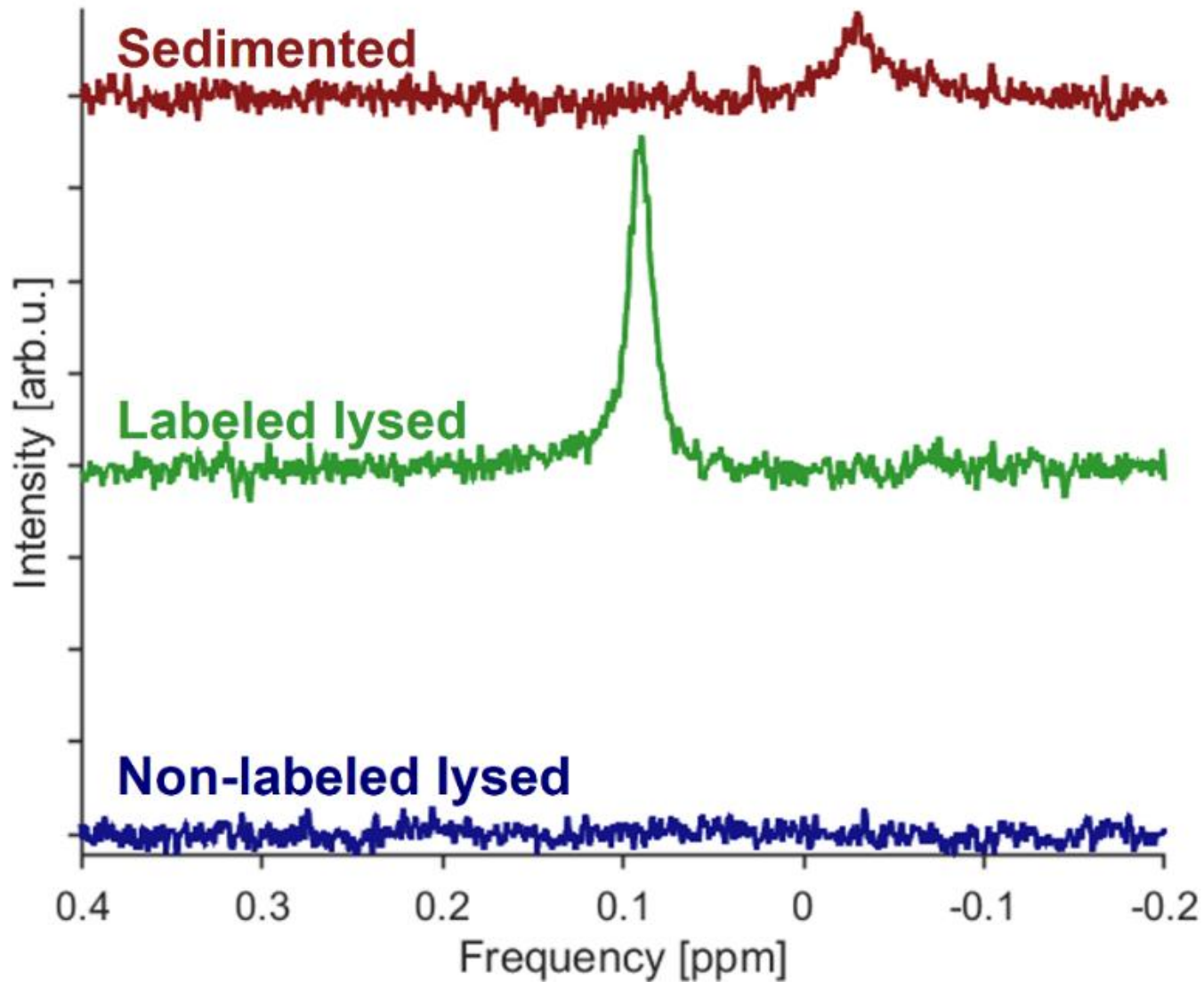

Figure S26. ^{1}H NMR with presaturation of the water signal, top: labeled cells following sedimentation, middle: labeled cells after cell lysis, bottom: non-labeled cells after cell lysis.

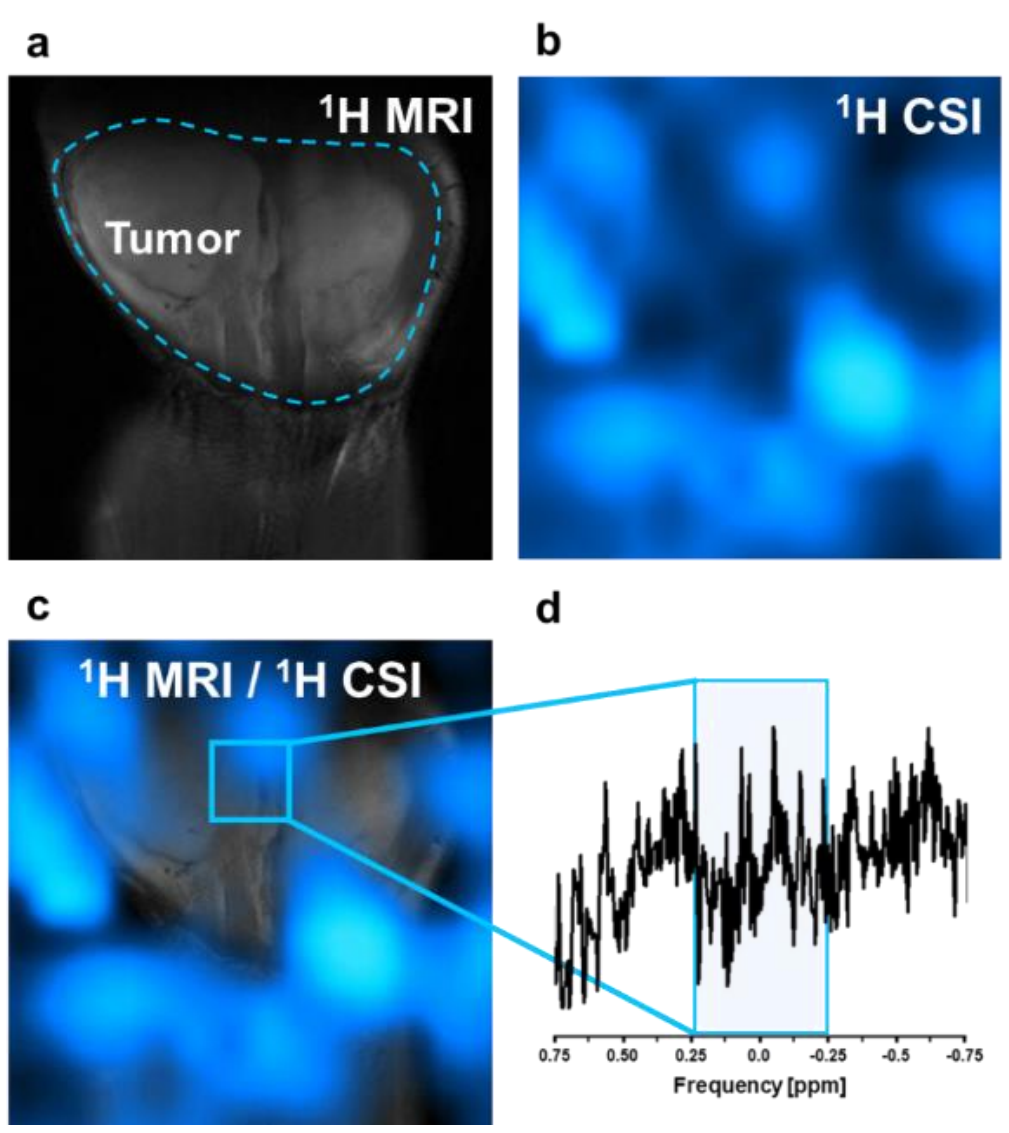

Figure S27. Absence of intrinsic 0 ppm signal in tumor-bearing mice prior to SiGHT administration. **a)** reference MRI image showing the tumor **b)** ^{1}H CSI image selected at 0 ppm range **c)** overlay of ^{1}H MRI and CSI shows no signal originating from tumor **d)** spectrum confirming the absence of SiGHT in solid tumor tissue.

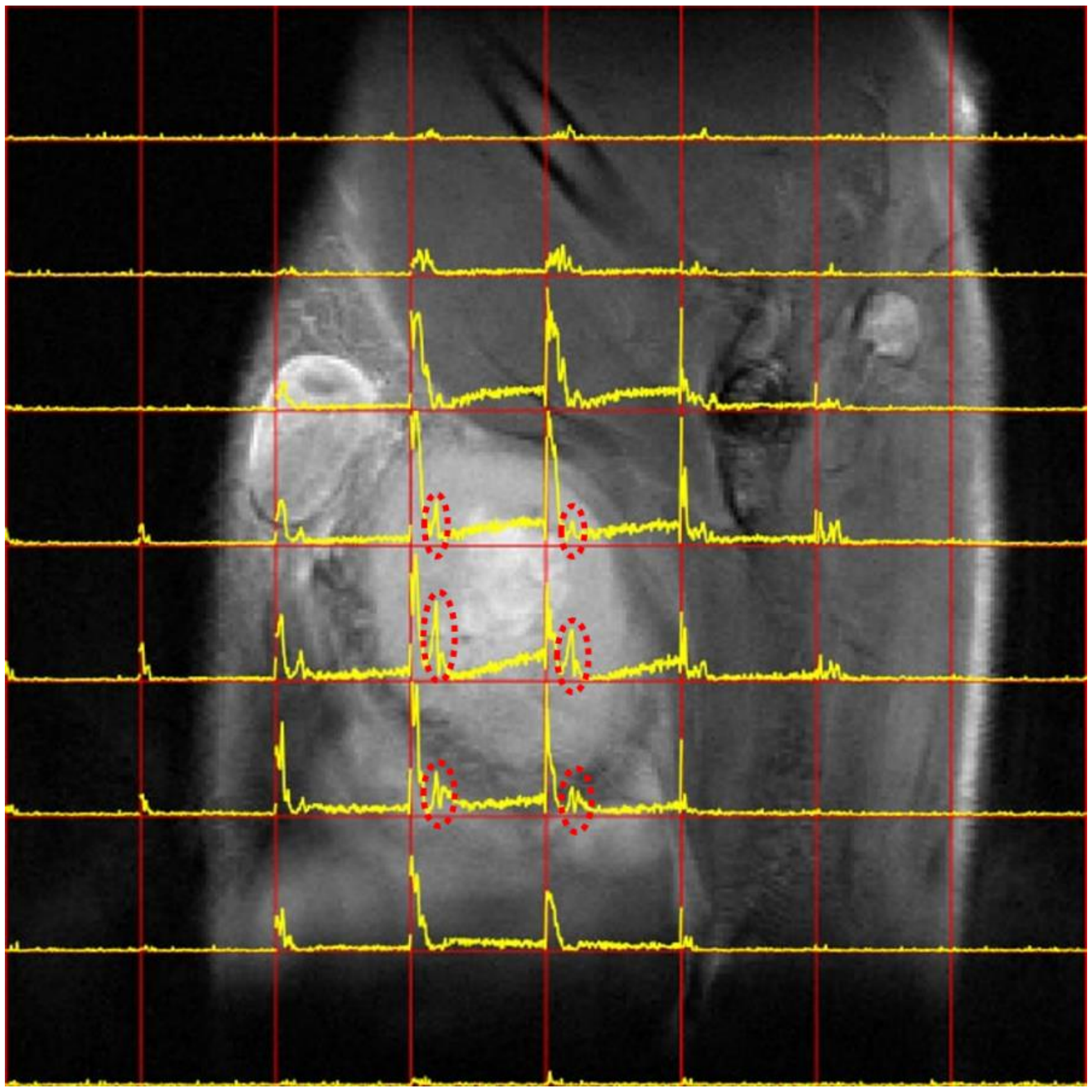

Figure S28. In vivo localized ^{1}H CSI spectral map of the solid tumor region. Standard anatomical ^{1}H MRI scan of a tumor-bearing animal flank, overlaid with a 2D CSI spectral grid. The localized yellow-colored MR spectra are displayed for each individual voxel (red grid cells). Red oval highlights the signal at ~0 ppm within the solid tumor corresponding to the SiGHT organosilicon tracer.

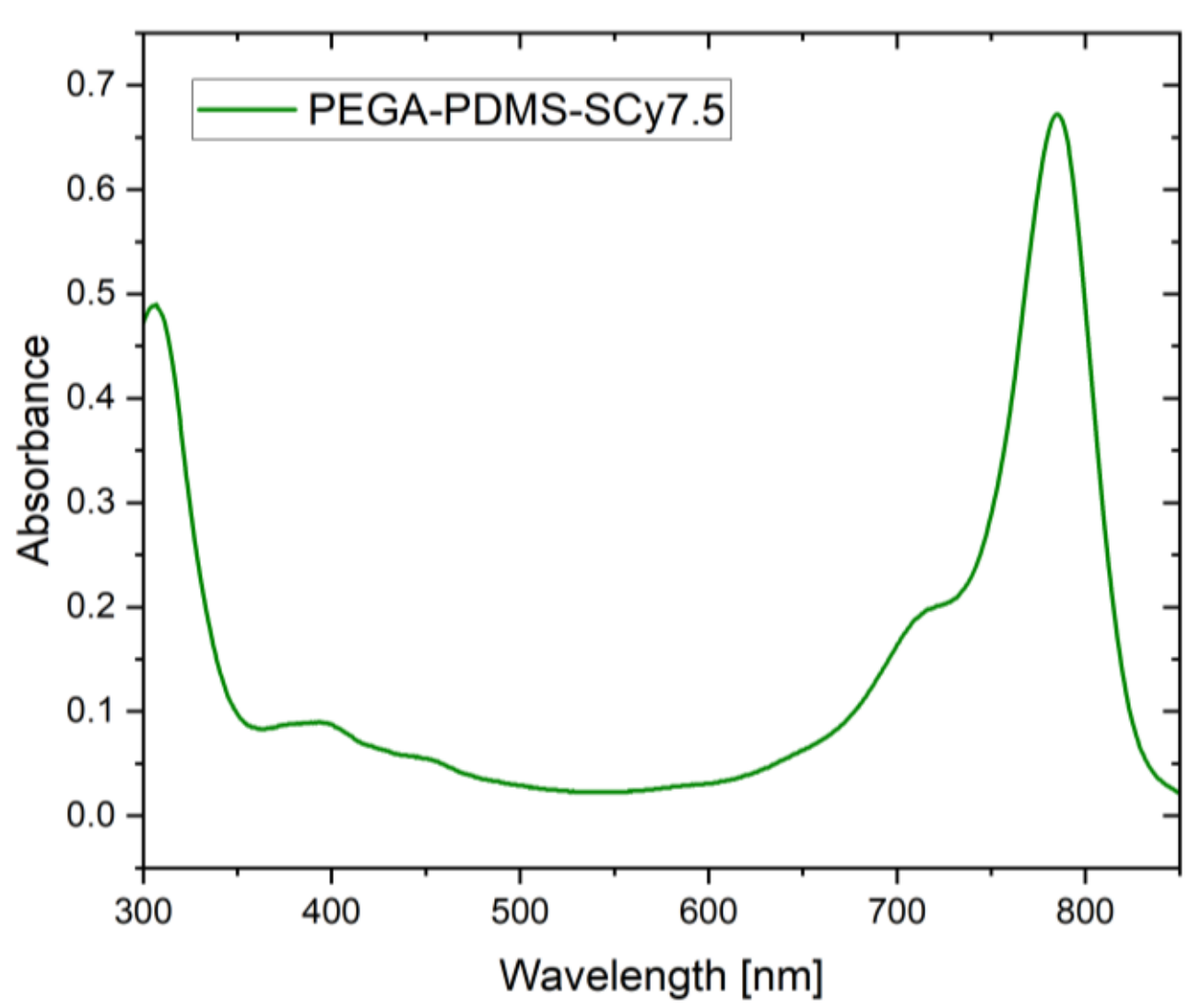

Figure S29. UV-Vis spectrum of POEGA-*co*-SCy7.5Am-*b*-PDMS after final purification (diluted to 0.5 mg mL^{-1} in water).

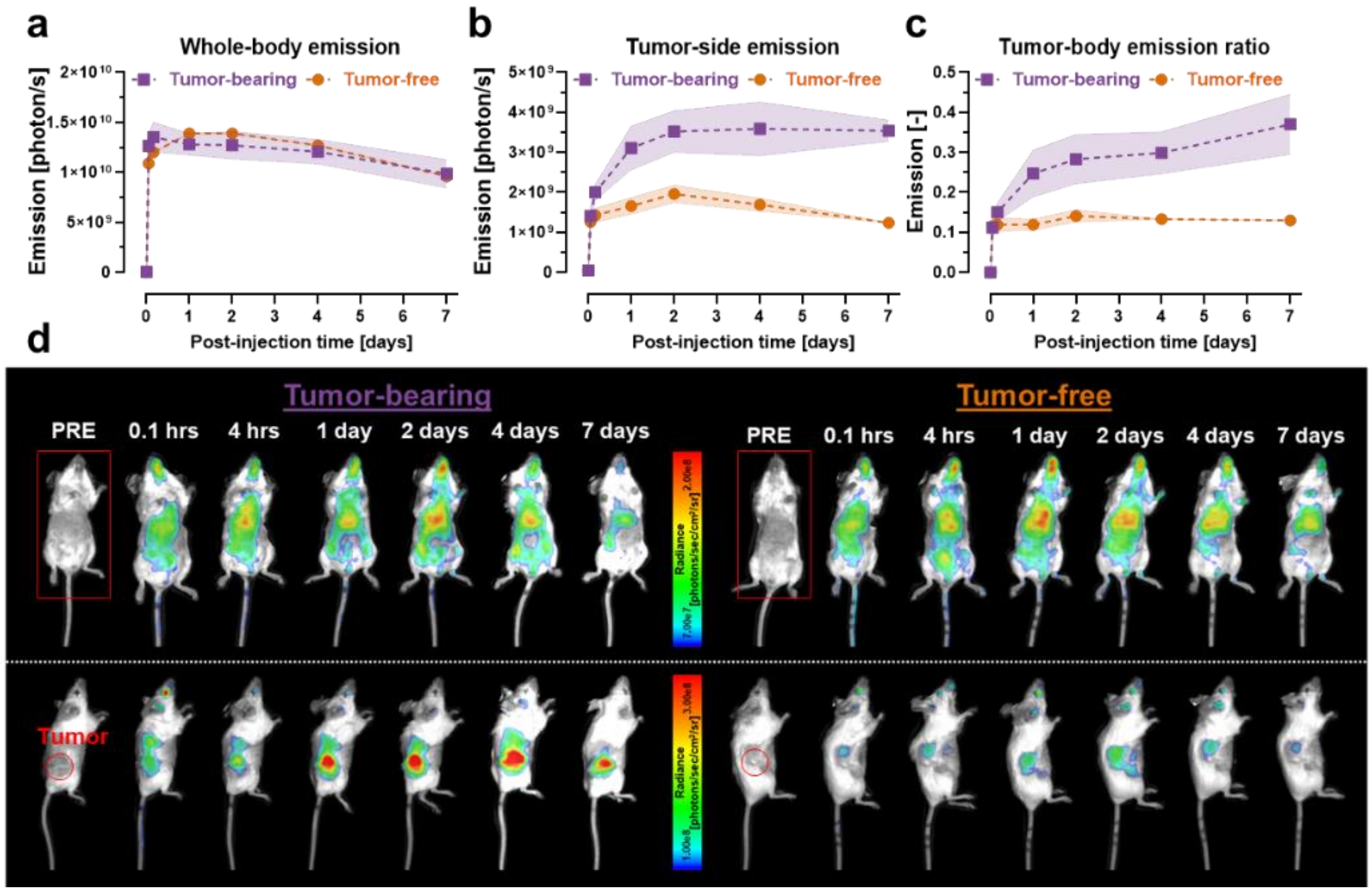

Figure S30. In vivo FLI biodistribution of SiGHT upon iv. administration (V = 100 uL, c = 50 mg mL-1). **a)** Whole-body emission shows rapid systemic dilution, followed by gradual clearance in both tumor-bearing and tumor-free groups. **b)** Tumor-region emission increases over time in tumor-bearing mice, plateauing around day 2, indicating accumulation and retention; tumor-free mice show a post-peak decline. **c)** Tumor-to-body emission ratio increases in tumor-bearing mice, while remaining constant in tumor-free controls, reflecting selective retention. Red outlines indicate ROIs used for quantification (n=3/group). **d)** Representative in vivo FLI images.

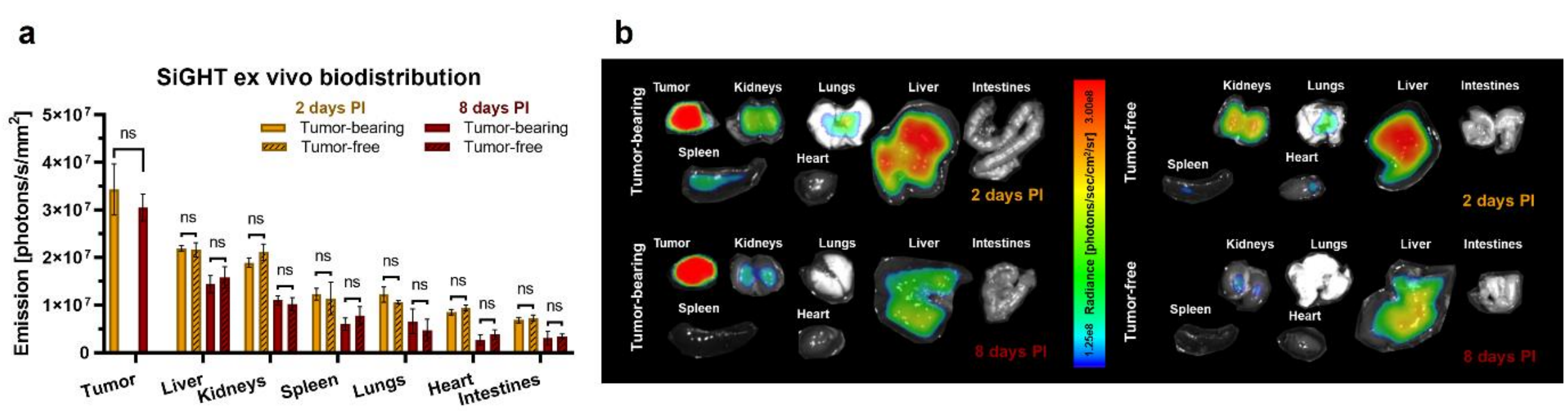

Figure S31. Ex vivo FLI biodistribution. **a)** Semi-quantitative FLI analysis reveals a statistically significant decrease in signal intensity at 8 days post-injection (PI) compared to 2 days PI. Notably, no significant differences in organ-associated fluorescence were observed between tumor-bearing and tumor-free groups. (n=3/group) **b)** Ex vivo representative FLI data.

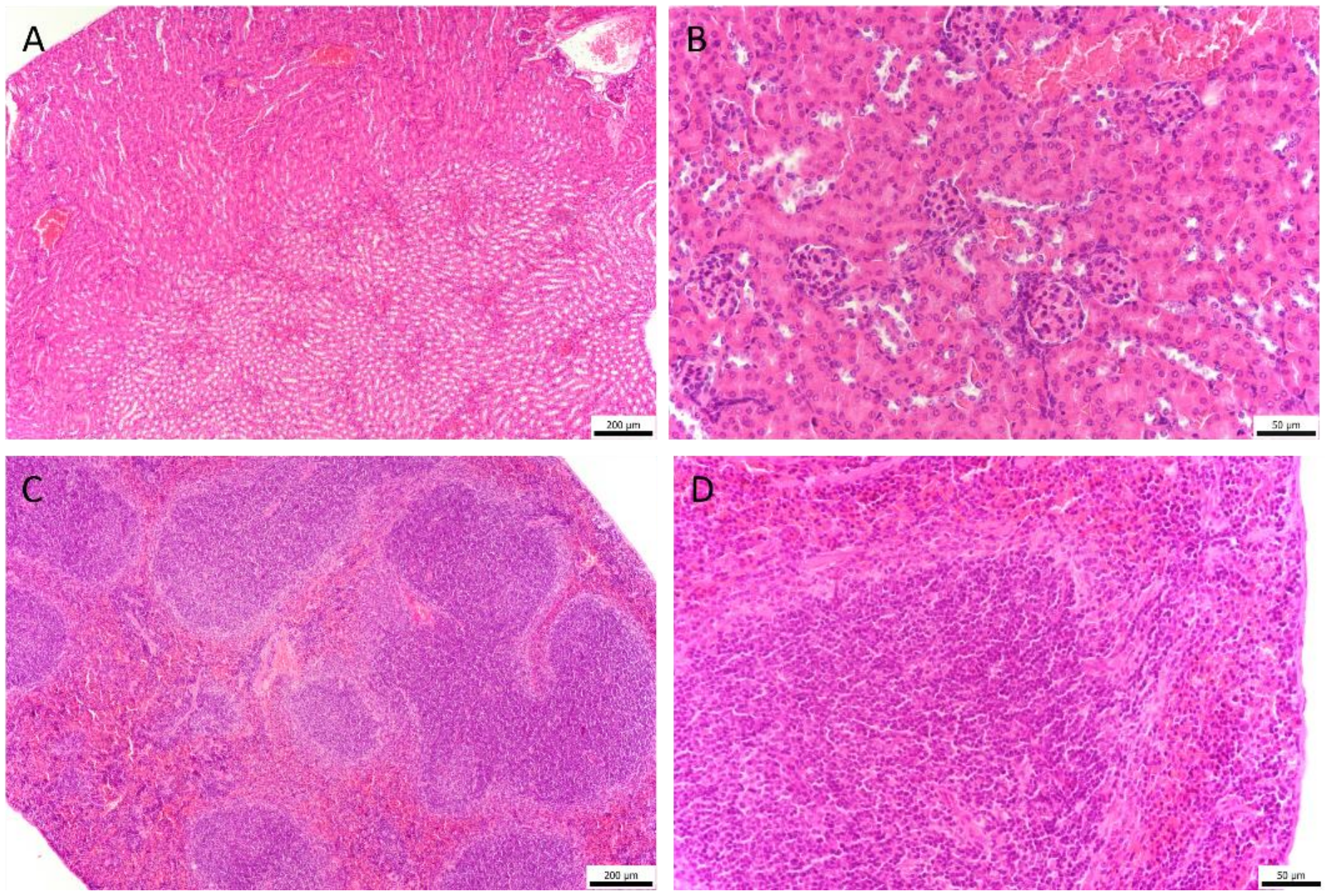

Figure S32. Representative histological images of organs from treated animals stained with hematoxylin and eosin. The upper row shows kidney morphology (A, B), and the lower row shows spleen morphology (C, D). Overall morphology and structure of both organs, as well as detailed images, exhibited normal tissue morphology and were free of any apparent pathological alterations.

References

[1] T. Lueckerath, T. Strauch, K. Koynov, C. Barner-Kowollik, D. Y. Ng, T. Weil, "DNA–polymer conjugates by photoinduced RAFT polymerization," *Biomacromolecules* **20** (2018), 20, 1: 212-221.

[2] A. Jerschow, N. Müller, *Vol. 125*, Academic Press, **1997**, pp. 372-375.

[3] D. N. Mastronarde, "Automated electron microscope tomography using robust prediction of specimen movements," *Journal of structural biology* **152** (2005), 152, 1: 36-51.
